# Autophagy role(s) in response to oncogenes and DNA replication stress

**DOI:** 10.1038/s41418-019-0403-9

**Published:** 2019-08-14

**Authors:** Riccardo Vanzo, Jirina Bartkova, Joanna Maria Merchut-Maya, Arnaldur Hall, Jan Bouchal, Lars Dyrskjøt, Lisa B. Frankel, Vassilis Gorgoulis, Apolinar Maya-Mendoza, Marja Jäättelä, Jiri Bartek

**Affiliations:** 1 Danish Cancer Society Research Center, Copenhagen, Denmark; 2 Department of Medical Biochemistry and Biophysics, Division of Genome Biology, Science for Life Laboratory, Karolinska Institute, Stockholm, Sweden; 3 Department of Clinical and Molecular Pathology, Institute of Molecular and Translational Medicine, Faculty of Medicine and Dentistry, Palacky University, Olomouc, Czech Republic; 4 Department of Molecular Medicine, Aarhus University Hospital, Aarhus, Denmark; 5 Biotech Research and Innovation Centre, University of Copenhagen, Copenhagen, Denmark; 6 Department of Histology and Embryology, School of Medicine, National Kapodistrian University of Athens, Athens, Greece; 7 Biomedical Research Foundation of the Academy of Athens, Athens, Greece; 8 Faculty Institute of Cancer Sciences, University of Manchester, Manchester Academic Health Science Centre, Manchester, UK

**Keywords:** Oncogenes, Macroautophagy

## Abstract

Autophagy is an evolutionarily conserved process that captures aberrant intracellular proteins and/or damaged organelles for delivery to lysosomes, with implications for cellular and organismal homeostasis, aging and diverse pathologies, including cancer. During cancer development, autophagy may play both tumour-supporting and tumour-suppressing roles. Any relationships of autophagy to the established oncogene-induced replication stress (RS) and the ensuing DNA damage response (DDR)-mediated anti-cancer barrier in early tumorigenesis remain to be elucidated. Here, assessing potential links between autophagy, RS and DDR, we found that autophagy is enhanced in both early and advanced stages of human urinary bladder and prostate tumorigenesis. Furthermore, a high-content, single-cell-level microscopy analysis of human cellular models exposed to diverse genotoxic insults showed that autophagy is enhanced in cells that experienced robust DNA damage, independently of the cell-cycle position. Oncogene- and drug-induced RS triggered first DDR and later autophagy. Unexpectedly, genetic inactivation of autophagy resulted in RS, despite cellular retention of functional mitochondria and normal ROS levels. Moreover, recovery from experimentally induced RS required autophagy to support DNA synthesis. Consistently, RS due to the absence of autophagy could be partly alleviated by exogenous supply of deoxynucleosides. Our results highlight the importance of autophagy for DNA synthesis, suggesting that autophagy may support cancer progression, at least in part, by facilitating tumour cell survival and fitness under replication stress, a feature shared by most malignancies. These findings have implications for better understanding of the role of autophagy in tumorigenesis, as well as for attempts to manipulate autophagy as an anti-tumour therapeutic strategy.

## Introduction

Macroautophagy (hereafter referred to as autophagy) is a highly conserved “self-eating” process, in which, under growth-unfavourable conditions, portions of the cytoplasm and/or intracellular organelles are engulfed in characteristic double-membrane structures (autophagosomes) that subsequently fuse with lysosomes [1]. This results in the degradation and recycling of intracellular components [2]. Autophagy is activated in response to diverse cellular stressors, such as increased levels of reactive oxygen species (ROS), starvation or DNA damage [3]. So far, 42 autophagy-related genes (*ATG*) have been identified [4]. The initiation step in nascent autophagic vesicle formation requires the activity of the ULK complex. The nucleation event, which follows initiation, is driven by the Vps34 complex. ATG7 and ATG10 enzymes mediate covalent attachment of ATG12–ATG5. The ATG5–ATG12 conjugate in a complex with ATG16 helps in the lipidation of ATG8 (LC3B) proteins, which are required for the elongation of the autophagosomal membrane. Following completion, the autophagic vesicle fuses with a lysosome to form a mature autolysosome [3]. p62 (SQTM1 in humans) is an autophagy-related, ubiquitin-binding protein whose short LC3B-interacting region facilitates a direct binding to LC3B, thereby inducing p62 degradation by autophagy [5]. In a context-dependent manner, p62 levels increase under autophagy inhibition and decrease when autophagy is induced, therefore, p62 can conveniently be used to study autophagic flux [6]. On the other hand, oncogene induction can lead to p62 overexpression [7], hence enhanced abundance of p62 in cancer cells does not necessarily indicate a blockade of autophagy. The formation of LC3B-positive puncta, monitored by immunodetection or fluorescent tagging, is a commonly used biomarker of autophagy induction. Furthermore, LC3B turnover represents yet another indicator of active autophagy and can be assessed by immunoblotting [8].

While a link of autophagy with genome maintenance and DNA repair has been suggested in that defective autophagy undermines homologous recombination, the exact mechanism behind this connection remains a matter of debate [9, 10]. Autophagy has been suggested to suppress tumour growth during early stages of cancer development [11, 12]. Conversely, autophagy can also promote tumour growth, for example by alleviating metabolic stress after chemotherapy [13] or by fuelling progression of early lesions to aggressive tumours in murine models [14]. Overall, the role of autophagy in cancer appears to be complex, context-dependent and currently incompletely understood [15].

Deregulated oncogenes cause replication stress (RS) and DNA damage in cultured cells and mouse models [16], thereby triggering DNA damage response (DDR) checkpoints that provide a biological anti-cancer barrier in early stages of tumorigenesis, a concept shared by responses to diverse types of oncogenes, loss of some tumour suppressors, and supported by analyses of clinical samples from a wide range of human cancer types [17, 18]. Indeed, preneoplastic and early cancerous lesions show features of RS, preferential DNA breakage in the difficult-to-replicate genomic fragile sites, and markers of constitutively active DNA damage checkpoint signalling [18], consistently with the notion that RS is one of the major sources of DNA damage during tumorigenesis [19]. Oncogenic activation and ensuing RS commonly evoke oncogene-induced senescence (OIS) or death of the incipient cancer cells, at the same time providing a context with selective pressure for outgrowth of cancer cells with defective DDR checkpoints, such as those with mutations in the ATM-Chk2-p53 axis [20]. Closely relevant to the topic addressed in our present study, RAS oncogene activation induces both RS [21] and autophagy [22]. In RAS-induced transformation (and more generally in response to oncogenic stress), autophagy might be activated to eliminate tumour cells or to limit proliferation of such potentially hazardous cells, by contributing to induction of senescence [23]. Alternatively, however, oncogenic RAS could induce autophagy to deal with metabolic stress and to promote tumour survival [24]. Ras mutations are commonly detected in different tumour types, including human urinary bladder and prostate cancers [25].

Given the intriguing, yet currently not fully understood, relationship between autophagy and RS, we have designed this study to address some of the outstanding issues in this field, both in response to oncogenes and genotoxic insults, such as those used in cancer treatment. Our dataset is based on complementary analyses of human clinical specimens from diverse stages of cancer progression, as well as human cellular models of oncogene- and drug-induced RS. Among the questions we address are the following: (i) Is the DDR checkpoint or autophagy activated first during natural human tumorigenesis and upon induction of oncogenic stress in cultured cells? (ii) What are, if any, mutual functional dependencies between RS/DDR and autophagy? (iii) Mechanistically, does autophagy impact the function of DNA replication forks under normal and/or RS conditions, and what are the potential implications of such RS-autophagy interplay? The answers to these questions are presented below, overall illustrating the order of events in response to oncogenic stress and evidencing a new role of autophagy in genome integrity maintenance, DNA replication and fork recovery from RS/DNA damage, with implications for cancer biology and treatment.

## Results

### Autophagy in clinical tumour samples and oncogene-expressing cells

**Fig. 1:**
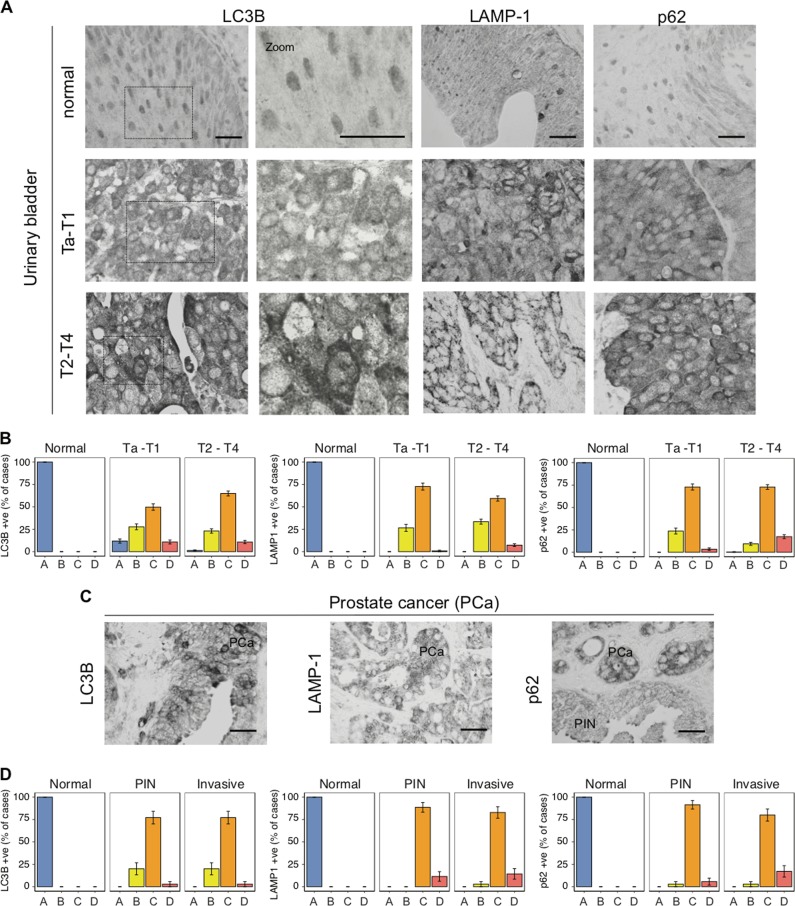
Autophagy patterns in clinical cancer specimens and oncogene-driven cellular models. **a** Representative immunohistochemistry images of normal bladder mucosa (normal), early lesions (Ta–T1) and advanced bladder carcinomas (T2–T4). Note: dominant cytoplasmic staining for LC3B, LAMP-1, and both cytoplasmic and nuclear localization of p62 [6, 70]. Scale bars, 50 µm. **b** Subdivision of immunohistochemistry results for LC3B, p62 (cytoplasmic) and LAMP-1 into four classes according to staining patterns: A being the lowest and D the highest degree of positivity. Mean and SD are indicated for the estimated frequency in each class (*N* > 300). **c** Representative examples of immunohistochemical patterns in prostate cancers. PCa: prostate cancer, PIN: prostatic intraepithelial neoplasia. Sections were stained for LC3B, LAMP-1 or p62. Scale bars, 50 µm. **d** Subdivision of immunohistochemistry results for LC3B, p62 and LAMP-1 into four classes according to the staining patterns: A being the lowest and D the highest degree of positivity. Mean and SD are indicated for the estimated frequency in each class (*N* = 35)

To determine whether autophagy induction is a common event in early human tumour lesions and to what extent the autophagy level changes during tumour progression, we compared early lesions (stages Ta–T1, *n* = 195) and more advanced stages (T2–T4, *n* = 308) of human urinary bladder cancer. In addition, a cohort of 35 cases of human clinical prostate specimens, each represented by progressing lesions, from normal tissue to prostatic intraepithelial neoplasia (PIN), up to invasive prostate carcinoma (PCa) was also examined. Notably, prior to biopsy sampling, neither the bladder nor prostate cancer patients received radiation or chemotherapy. Using well validated markers and the established sensitive immunoperoxidase protocol [26, 27], we consistently detected an increase in the level of cytoplasmic, dot-like signal for LC3B and p62 in both early and late lesions, as compared with normal tissue (Figs. 1a–d and S1). Moreover, the lysosomal marker LAMP-1 showed an increased granular staining pattern in early and late lesions, suggesting that lysosomal degradation of autophagy cargoes might be induced relatively early among human epithelial tumours compared with the corresponding normal tissues.

In our previous study, we found a strong activation of DDR checkpoint signalling in Ta–T1 bladder lesions, which was then partly diminished in T2–T4 stages [17]. Notably, we did not find such decrease of any of the autophagy-associated markers in advanced tumours, as we did not observe any major differences in early vs advanced lesions of either urinary bladder or prostate cancer (Figs. 1a–d and S1A, B). Our results are compatible with a model, in which the process of autophagy is enhanced already at the early stages of human tumour development, and autophagy is maintained also at the invasive carcinoma stages, for both urinary bladder and prostate tumours.

**Fig. 2:**
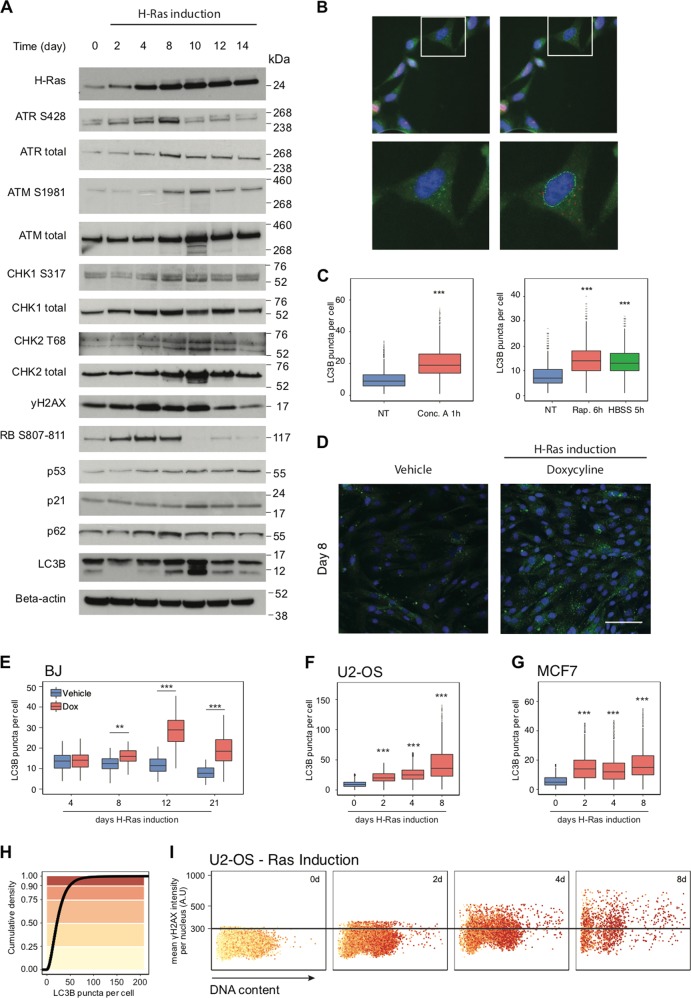
Autophagosomes accumulate upon overexpression of H-RasV12. **a** The level of DNA damage response- and autophagy-related proteins was tested by immunoblotting in BJ-Ras cells. Cells were treated with 2 μg/ml of doxycycline (Dox) for the indicated time. Actin was used as a loading control. **b** Representative images of LC3B puncta and nuclear counterstaining (DAPI). Bottom, cropped images from the top. **c** Quantification of LC3B puncta per cell in U2-OS cells treated with 2 nM of concanamycin A (cells analyzed per condition > 5000), 100 nM of rapamycin or maintained in HBSS for the indicated time (cells analyzed per condition > 1000). *P* value associated to two-sided *t*-test for the difference to the non-treated control. **d** Representative images of LC3B puncta and nuclear counterstaining (DAPI) in BJ fibroblasts incubated with DMSO (vehicle) or 2 μg/ml of doxycycline (Dox) for 8 days. Scale bars, 100 µm. **e** Quantification of LC3B puncta per cell in BJ fibroblasts incubated with DMSO (vehicle) or 2 μg/ml of doxycycline (Dox) for the indicated days. Pictures analyzed per condition > 200, at least 20 cells per picture. *P* value associated to two-sided *t*-test for the difference to the matched control. **f** Quantification of LC3B puncta per cell in U2-OS cells incubated with 2 μg/ml of doxycycline (Dox) for the indicated days. Cells analyzed per condition > 1500. *P* value associated to two-sided *t*-test for the difference to the untreated control. **g** Quantification of LC3B puncta per cell in MCF7 cells incubated with 2 μg/ml of doxycycline (Dox) for the indicated time. Cells analyzed per condition > 2000. *P* value associated to two-sided *t*-test for the difference to the untreated control. **h** Cumulative density distribution of LC3B puncta per cell from the experiment in **f** is shown. Colours correspond to the colour code used in a single-cell analysis. **i** A single-cell analysis (single points on the scattering plot) of γH2AX mean nuclear intensity (*y*-axis), LC3B puncta per cell (colour code from (**h**)) and DNA content (*x*-axis) from the experiment in **f**

While providing valuable evidence for the clinical relevance, the immunohistochemical analysis of tumour sections collected at a certain time point during the lengthy period of disease progression does not allow to establish a more precise timing of critical events, in this case particularly whether autophagy or DDR checkpoints are triggered first in tumorigenesis. To experimentally address the latter issue, and gain further insights into the contribution of autophagy to tumorigenesis, we ectopically expressed a constitutively active version of the H-Ras oncogene, H-RasV^12^ (referred to as RAS) under the control of a Tet-dependent promoter in different cell types. We induced RAS expression and evaluated DDR and autophagy parameters in parallel over time. Using immunoblotting, we detected initial DDR activation between days 2–4 of RAS induction and culmination by day 8, while autophagy markers accumulated later, from day 8 (Fig. 2a). Similarly to the immunohistochemical analysis, we observed accumulation of p62 over time, however, autophagy flux was not compromised by RAS overexpression (Fig. S1C). To monitor the number of autophagosomes, we detected LC3B puncta by immunofluorescence [28] and adapted a high-content image analysis method to analyze the LC3B-labelled vesicles in the cytoplasm (Fig. 2b). As positive controls to validate our analysis, we increased LC3B puncta accumulation by treating cells with either concanamycin A (inhibits autophagy and prevents LC3B puncta disappearance), rapamycin (the mTORC1 inhibitor that blocks mTORC1-mediated autophagy inhibition, induces autophagy and increases LC3B puncta formation) or starvation (Fig. 2c). Using this innovative high-content approach, we could concomitantly monitor and correlate the number of LC3B puncta, DDR signalling markers and the cell-cycle position at a single-cell level. RAS overexpression induced autophagy before the establishment of OIS [29] in all cellular systems we have tested (Fig. 2d–g). Importantly, RAS induction did not compromise autophagy flux (Fig. S2A–C). While LC3B puncta correlated with the time of oncogene induction, at the single-cell level we did not observe any clear-cut correlation between LC3B puncta and the level of the well-established DDR marker, γH2AX. Instead, we noticed accumulation of LC3B puncta in cells in late S and G2 cell-cycle phases (Figs. 2h, i and S2D). An early event upon oncogenic activation is accumulation of RS, an emerging hallmark of cancer [18, 21]. RS reflects insults that negatively affect DNA replication, commonly resulting in impaired, arrested or collapsed replication forks [20]. Several mechanisms have been proposed to explain how oncogenes induce RS. Cyclin E overexpression enhances collisions between transcription and replication machineries [30], Cdc6 overexpression results in excessive origin firing and slow fork progression [18], while c-Myc overexpression alters the transcription programme and, ultimately, affects metabolism and dNTP levels [21, 31]. In our experiments, cyclin E and c-Myc overexpression also induced γH2AX accumulation and LC3B puncta, with no clear correlation between these two parameters at the single-cell level (Fig. S3). Together, and consistently with another report [32], we showed that oncogenic expression induced autophagy independently of the cell type, while the strength of DDR signalling and precise kinetics of autophagy induction were oncogene- and cell type-dependent, with an overall trend for the DDR activation to precede autophagy induction.

### DNA replication stress induces autophagy

**Fig. 3:**
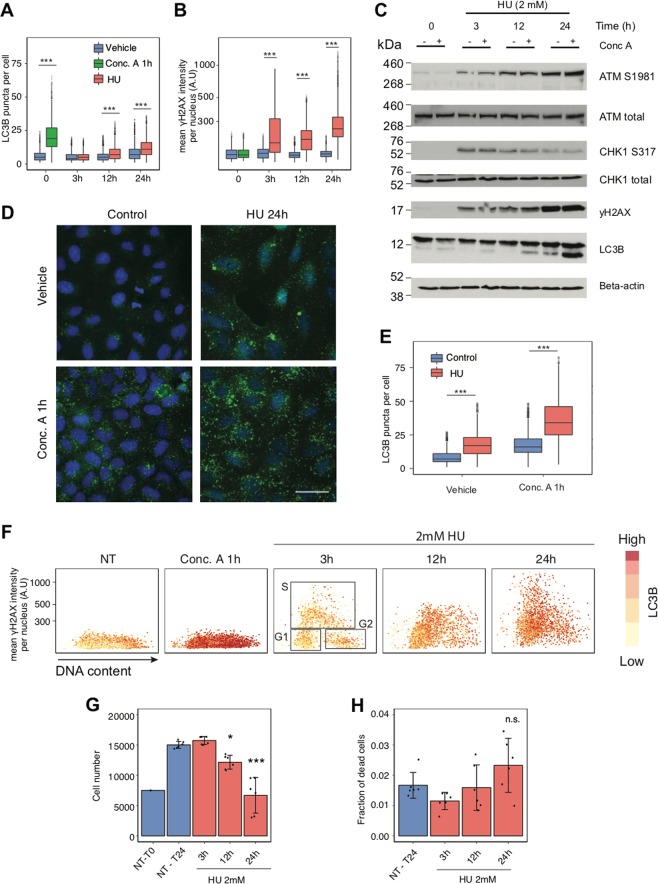
DNA replication stress induces autophagy. **a** Quantification of LC3B puncta per cell in U2-OS cells incubated with DMSO (vehicle) or 2 mM of hydroxyurea (HU) for the indicated time. As a positive control of an increase in the amount of autophagosomes per cell, U2-OS cells were treated for 1 h with 2 nM of concanamycin A (Conc. A). Cells analyzed per condition > 2500. *P* value associated to two-sided *t*-test for the difference to the matched control. **b** γH2AX mean nuclear intensity in a single-cell analysis, with experimental conditions as in **a**. *P* value associated to two-sided *t*-test for the difference to the matched control. **c** The level of DNA damage response- and autophagy-related proteins was tested by immunoblotting in U2-OS cells. Cells were treated with 2 mM of hydroxyurea (HU) for the indicated time. Where indicated, cells were incubated with 2 nM of concanamycin A (Conc. A) for 1 h prior to harvesting. Actin was used as a loading control. **d** Representative images of LC3B puncta and nuclear counterstaining (DAPI) in U2-OS. Cells were incubated with 2 mM of hydroxyurea (HU) for 24 h and/or 2 nM of concanamycin A (Conc. A) for 1 h prior to fixation, where indicated. Scale bars, 50 µm. **e** Quantification of LC3B puncta per cell in untreated (control) U2-OS cells or cells treated with 2 mM of hydroxyurea (HU) for 24 h. Where indicated, cells were incubated with 2 nM of concanamycin A (Conc. A) for 1 h prior to fixation. Cells analyzed per condition > 2500. *P* value associated to two-sided *t*-test for the difference to the matched control. **f** A single-cell analysis of γH2AX mean nuclear intensity, LC3B puncta per cell and DNA content in U2-OS cells, with experimental conditions as in **a**. Gates for the cell cycle phases are shown in HU 3 h. **g** Quantification of U2-OS cells incubated with 2 mM of hydroxyurea (HU) for the indicated time. Error bars indicate mean and SD for each independent biological replicate (*N* = 6). *P* value associated to two-sided *t*-test for the difference to the untreated control. **h** Quantification of the fraction of dead U2-OS cells from (**g**). Error bars indicate mean and SD for each independent biological replicate (*N* = 6)

To corroborate the notion that RS induces autophagy, we used different drugs known to induce RS through direct or indirect mechanisms [33]. Hydroxyurea (HU), which has been commonly applied under laboratory conditions to stall and arrest replication forks, binds to the M2 subunit of the ribonucleotide reductase (RRM) and inhibits its activity. RRM reduces ribonucleotides to provide deoxyribonucleotides needed for DNA synthesis [34]. It has been shown that short, 30-min incubation with HU induces fork stalling, but after drug removal, forks restart DNA synthesis. In contrast, exposure of cells to HU for 3–4 h results in collapsed forks that need to be rescued by firing new replication origins [35]. We performed a time course experiment to investigate whether HU treatment induces autophagy, at the same time monitoring the dynamics of DDR activation. Cells triggered autophagy 12 h after treatment with 2 mM of HU, while strong accumulation of γH2AX was seen already after 3 h of HU exposure (Fig. 3a–c). HU did not compromise autophagy flux and, as expected, cells accumulated DNA damage during S phase (Fig. 3d–f). We also observed that the high level of autophagy correlated with impaired cell proliferation after 24 h of HU treatment (Fig. 3g, h). Furthermore, no correlation was observed between the level of γH2AX and LC3 puncta after 24 h in the presence of HU (analysis of correlation = 0.18).

**Fig. 4:**
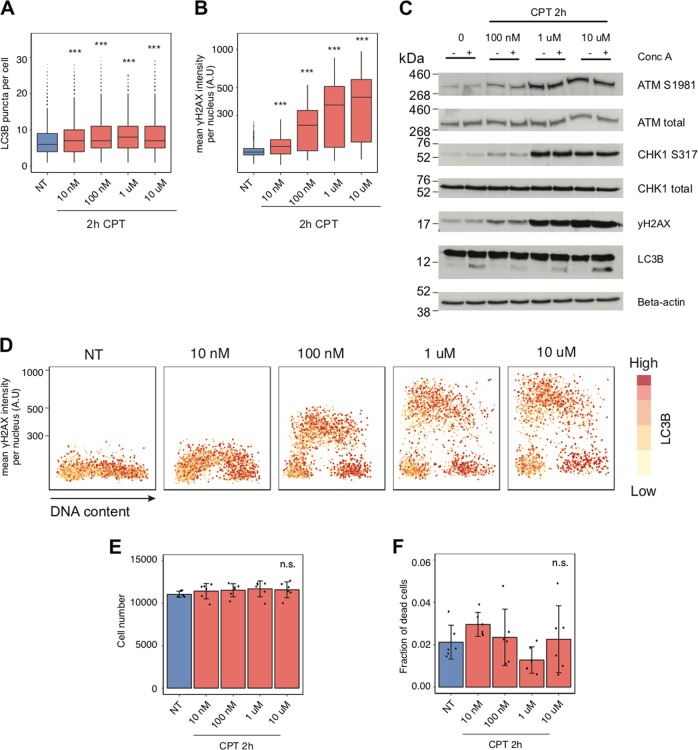
Camptothecin treatment and autophagy. **a** Quantification of LC3B puncta per cell in U2-OS cells incubated with different concentrations of camptothecin (CPT) for 2 h. Cells analyzed per condition > 2500. *P* value associated to two-sided *t*-test for the difference to the untreated control. **b** γH2AX mean nuclear intensity in a single-cell analysis, with experimental conditions as in **a**. **c** The level of DNA damage response- and autophagy-related proteins was tested by immunoblotting in U2-OS cells. Cells were incubated with different concentrations of camptothecin (CPT) for 2 h. Where indicated, cells were incubated with 2 nM of concanamycin A (Conc. A) for 1 h prior to harvesting. Actin was used as a loading control. **d** A single-cell analysis of γH2AX mean nuclear intensity, LC3B puncta per cell and DNA content from (**a**, **b**). **e** Quantification of U2-OS cells treated with different concentrations of camptothecin (CPT) for 2 h. Error bars indicate mean and SD for each independent biological replicate (*N* = 6). **f** Quantification of the fraction of dead U2-OS cells from **e**. Error bars indicate mean and SD for each independent biological replicate (*N* = 6)

Next, we tested the effect of the topoisomerase I inhibitor, camptothecin (CPT). CPT modestly induced autophagy after 2 h and robustly triggered DDR activity, without compromising cell viability (Figs. 4 and S4A–E). In cells treated with CPT for 2 h at 10 µM, we did not observe any correlation at the single-cell level between γH2AX intensity and LC3B puncta (analysis of correlation = 0.16). Results of the 24 h treatments with cisplatin or aphidicolin, drugs that also induce RS, followed the same trend, DDR was activated first and autophagy later (Fig. S4F–J).

**Fig. 5:**
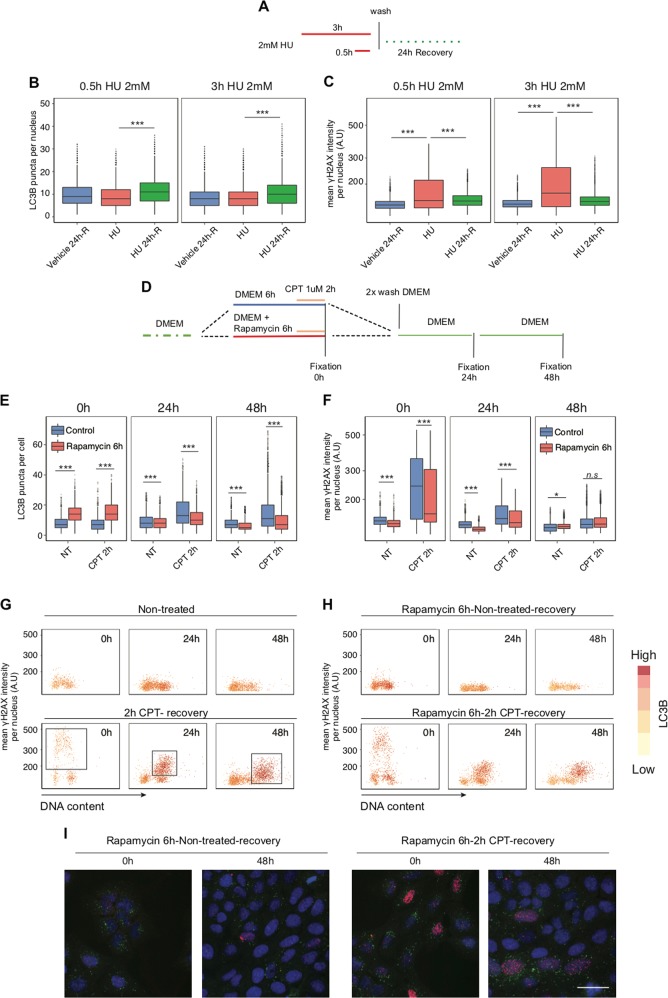
The relationship between drug-induced replication stress, DNA repair and autophagy. **a** The diagram of experimental settings for **b, c** is shown. **b** Quantification of LC3B puncta per cell in U2-OS cells that were incubated with 2 mM of HU for the indicated time, washed and left to recover from the drug for 24 h. Cells analyzed per condition > 2500. *P* value associated to two-sided *t*-test for the difference to the sample treated with 2 mM of HU for 2 h. **c** γH2AX mean nuclear intensity in a single-cell analysis, with experimental conditions as in **a**. Cells analyzed per condition > 2500. *P* value associated to two-sided *t*-test for the difference to the sample treated with 2 mM of HU for 2 h. **d** The diagram of experimental settings for (**e–h**) is shown. **e** Quantification of LC3B puncta per cell in U2-OS cells treated as in **d**. Cells analyzed per condition > 2500. **f** γH2AX mean nuclear intensity in a single-cell analysis, with experimental conditions as in **d**. **g** A single-cell analysis of γH2AX mean nuclear intensity, LC3B puncta per cell and DNA content in cells treated as in **d**. Cells analyzed per condition > 2500. Squares indicate the proportion of cells in S phase that accumulate a high level of autophagy (CPT 2 h, 1016 cells; 24 h recovery, 1178 cells; 48 h recovery, 879 cells). **h** Cells were pretreated with 100 nM of rapamycin for 6 h to induce autophagy prior to CPT treatment and analyzed as in **g**. **i** Representative images of LC3B puncta, γH2AX staining and nuclear counterstaining (DAPI) in U2-OS treated as in **h**. Scale bars, 50 µm

We then evaluated whether autophagy activity is diminished or augmented during recovery from transient RS, reasoning that if RS indeed induces autophagy, only the fraction of cells that experienced RS already during a short-term treatment with genotoxic drugs would induce autophagy during recovery from such RS pulse. To explore this rationale, we treated U2-OS cells with 2 mM of HU for either 0.5 or 3 h to induce arrested and collapsed forks, respectively, then changed the culture medium to remove HU (Fig. 5a) and allowed the cells to recover for 24 h, at which time the number of LC3B puncta and the intensity of γH2AX were quantified. We observed that the number of LC3B puncta was augmented during the recovery period, while the intensity of γH2AX was reduced, a result reproduced in two cell types (Figs. 5b, c and S5A, B). Thus, autophagy might not be activated as a primary response to RS. To test further the potential involvement of autophagy in RS and DDR, we incubated cells, either individually or concomitantly, with rapamycin and CPT, and allowed cells to recover for up to 48 h (Fig. 5d–f). Cells in S phase accumulated γH2AX when treated for 2 h with CPT. Interestingly, our single-cell analysis revealed that cells with a high level of γH2AX after CPT treatment showed also high amounts of LC3B puncta during recovery (Fig. 5g and squares, correlation analysis between LC3B puncta vs γH2AX intensity = 0.65, CPT-treated cells for 2 h and recovery 48 h). On the contrary, cells with low γH2AX after CPT incubation (cells in G1 phase) were also low for LC3B after 24–48 h of recovery. Regardless of their position in the cell cycle, rapamycin transiently induced LC3B puncta in every cell without increasing the γH2AX level. We hypothesized that if autophagy was activated as a response to RS, cells pretreated with rapamycin and not affected by CPT should not exhibit a high level of LC3B at recovery. Indeed, after recovery from CPT treatment, rapamycin-treated cells with low γH2AX were also characterized by low LC3B (Fig. 5h, i). LC3B puncta and the γH2AX level after CPT and recovery showed the same trend in a different cellular model (Fig. S5C–E). Together, our results show that, in a sequential order, DDR is triggered first upon RS and this is then followed by induction of autophagy. These observations raise the possibility that autophagy may be required for later stages of DNA repair to re-establish metabolic homoeostasis after DNA damage.

### Autophagy-deficient cells accumulate DNA replication stress

**Fig. 6:**
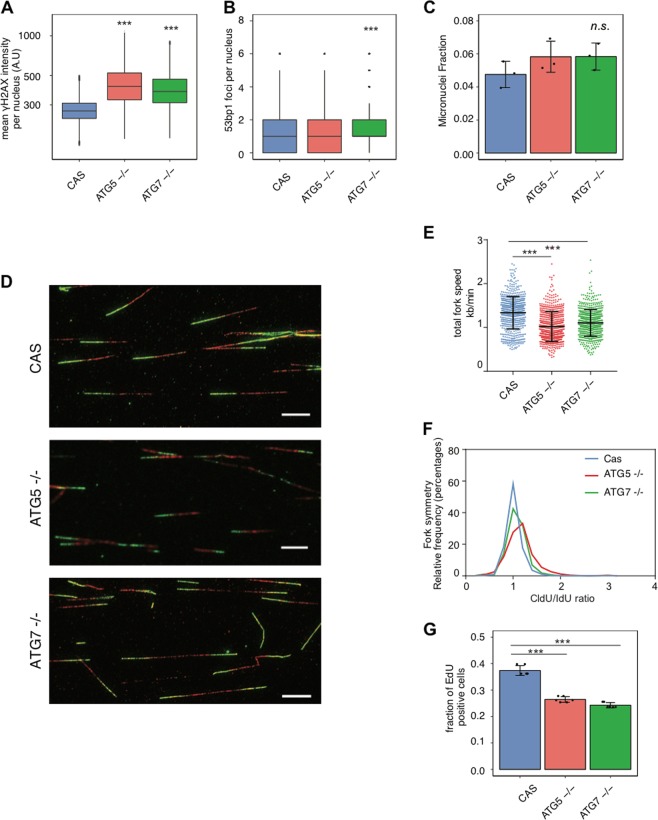
The relationship between drug-induced replication stress, DNA repair and autophagy. **a** γH2AX mean nuclear intensity per nucleus in knockout MCF7 cells: (CAS) parental control, *ATG5*−/− and *ATG7−*/−. Cells analyzed per condition > 16 000. **b** An average number of 53BP1 foci per nucleus in knockout MCF7 cells: (CAS) parental control, *ATG5*−/− and *ATG7−*/−. Cells analyzed per condition > 9000. *P* value associated to two-sided *t*-test for the difference to the matched control. **c** Micronuclei fraction in knockout MCF7 cells: (CAS) parental control, *ATG5−*/− and *ATG7−*/− (*N* = 3). *P* value associated to two-sided *t*-test for the difference to the matched control. **d** Examples of DNA fibres from knockout MCF7 cells. Scale bars, 10 µm. **e** Early passage MCF7 *ATG5*- and *ATG7*-knockout cells were pulse-labelled with CldU for 20 min, followed by a second pulse of IdU for 20 min. The length of CldU and IdU was measured and converted into fork speed in kb/min (results from three independent slides; scored forks CAS *N* = 646, average speed 1.33 kb/min; *ATG5−/−* *N* = 680, 1.0 kb/min; *ATG7−/−* *N* = 653, 1.1 kb/min). *P* value associated to two-sided *t*-test with Welch’s correction. **f** The ratio between CldU/IdU was analyzed and plotted as relative frequencies (CAS vs *ATG5−*/− *P* < 0.0001 Kolmogorov–Smirnov test; CAS vs *ATG7−/− P* < 0.0001 Kolmogorov–Smirnov test). **g** The proportion of knockout MCF7 cells in S phase (EdU positive). Mean and SD are plotted for six independent samples. *P* value associated to two-sided *t*-test for the difference to the matched control

Our results pointed to a potential role of autophagy in cell recovery from RS. We tested this possibility by knocking out two important autophagy genes, *ATG5* and *ATG7*, in two different human cellular backgrounds (Fig. S6A). Surprisingly, in both MCF7 and HeLa cells the absence of either of these genes resulted in accumulation of potentially RS-related DDR markers, such as γH2AX and 53BP1 foci and, to a lesser extent, in micronuclei formation (Figs. 6a–c and S6B–D). To measure RS directly at the level of DNA replication forks, we used the DNA fibre technique [36]. The absence of either *ATG5* or *ATG7* reduced the speed of fork elongation (Fig. 6d, e; fork speed: CAS control = 1.3 kb/min, *ATG5*−/− = 1.0 kb/min, *ATG7*−/− = 1.1 kb/min). Previous reports have shown that the drug-induced aberrant reduction of fork progression speed by 20% activates DDR and, potentially, affects genomic integrity [37, 38]. By measuring the symmetry of fork progression between both DNA-labelling pulses as a readout of stalled and collapsed forks [39], we detected an increase of asymmetric forks in the absence of autophagy genes (Fig. 6f; CAS vs *ATG5*−/− *P* < 0.0001, Kolmogorov–Smirnov test; CAS vs *ATG7−*/− *P* < 0.0001). Interestingly, the number of MCF7 knockout cells that entered S phase was decreased (Fig. 6g). These results show that in the absence of *ATG5* or *ATG7* cells experienced RS.

### Proficient mitochondrial function in the absence of *ATG5* and *ATG7*

**Fig. 7:**
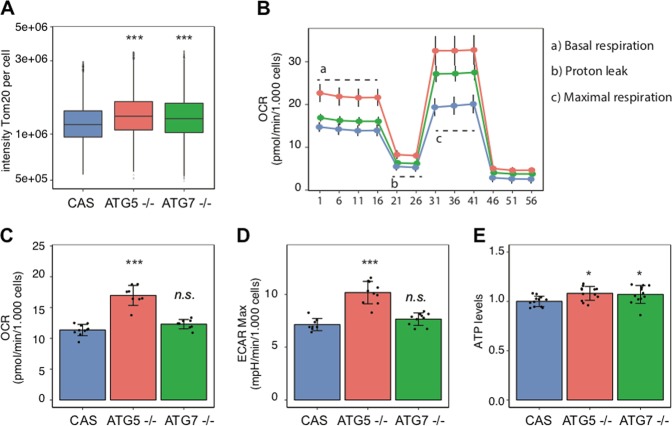
Knockout of autophagy genes has no detrimental effect on basal metabolism. **a** Tomm20 mean intensity per cell in knockout MCF7 cells: (CAS) parental control, *ATG5−*/− and *ATG7−*/−. Cells analyzed per condition > 10000. *P* value associated to two-sided *t*-test for the difference to the matched control. **b** Oxygen consumption rate (OCR) in *ATG5*- and *ATG7*-knockout MCF7 cells. Oligomycin (1 μM) was applied to inhibit the F_0_/F_1_-ATP synthase and evaluate the proton leak. Carbonylcyanide-p-trifluoromethoxyphenylhydrazone (FCCP) was added next to obtain the maximum respiratory rate (MRR). Finally, a combination of rotenone and antimycin-A was used to inhibit the activity of C-I and C-III and to measure non-mitochondrial respiration. Samples analyzed per cell line *N* = 8. **c** The mean of OCR values for knockout MCF7 cells. *P* value associated to two-sided *t*-test for the difference to the matched control. Samples analyzed per cell type *N* = 8. **d** The mean of ECAR values for knockout MCF7 cells. OCR and ECAR values were corrected for non-mitochondrial respiration. Samples analyzed per cell type *N* = 8. *P* value associated to two-sided *t*-test for the difference to the matched control. **e** The mean ATP level in knockout MCF7 cells (*N* = 12). *P* value associated to two-sided *t*-test for the difference to the matched control

Mitochondria are critical to maintain cellular bioenergetics and to regulate G1/S transition [40]. Mitophagy is a selective pathway to remove damaged, old or dysfunctional mitochondria through a process that, in most cases, requires autophagy [41]. Therefore, it is reasonable to assume that in the absence of *ATG5* or *ATG7* bioenergetics might be impaired due to defective mitochondrial function and impact the quality of genomic DNA replication. While we did not observe changes in the ROS levels in the absence of either *ATG5* or *ATG7*, cells were nevertheless more sensitive to hydrogen peroxide (Fig. S7A, B). Interestingly, we found that mitochondrial content was significantly higher in both *ATG5-* and *ATG7*-knockout cells (Figs. 7a and S7C). Furthermore, we applied a Seahorse XF analyzer to comprehensively examine the potential effect of the absence of *ATG5* and *ATG7* on cellular bioenergetics. We observed that mitochondrial respiration was enhanced in both *ATG5*−/− and *ATG7*−/− cells (Figs. 7b and S7D), although only *ATG5*-deficient cells showed significantly increased basal respiration (Figs. 7c and S7 E). Moreover, the maximum capacity of glycolysis (ECAR Max) was significantly higher in *ATG5*−/− cells (Figs. 7d and S7F). In parallel, intracellular ATP levels were increased in *ATG5*−/− and *ATG7*−/− MCF7 (Fig. 7e) but not HeLa cells (Fig. S7G), potentially reflecting the observed augmentation in bioenergetic processes. Thus, somewhat unexpectedly, our data show that mitochondria in both *ATG5-* and *ATG7*-depleted cells are operational, featuring functional parameters at levels either comparable with, or even higher than, those in the *ATG5/ATG7*-proficient counterpart cells.

### Autophagy and recovery from replication stress

Looking for additional evidence to explain how autophagy is related to RS, we focused our attention on the regulation of nucleotide levels. Given that induction of autophagy in some mouse models may positively impact nucleotide pools [42], we next investigated whether our autophagy-defective human cancer cell models are altered in their sensitivity to nucleotide depletion. First, we treated the *ATG5/7*-deficient cells for 3 h with HU to block dNTP synthesis. Some cells were fixed after HU incubation, while others were washed and grown in the full medium, allowing cells to recover from nucleotide depletion.

**Fig. 8:**
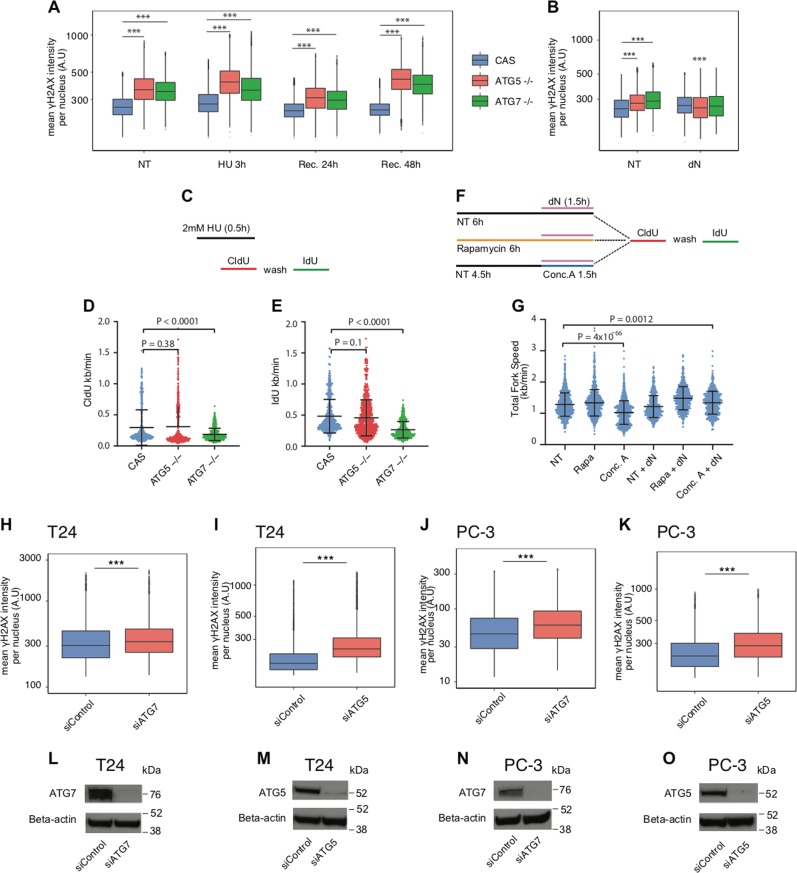
Autophagy is required for efficient recovery from RS. **a** γH2AX mean nuclear intensity in knockout MCF7 cells after treatment with 2 mM of HU for 3 h and during recovery. Cells analyzed per condition > 8000. *P* value associated to two-sided *t*-test for the difference to the matched control. **b** γH2AX mean nuclear intensity in knockout MCF7 cells after treatment with 100 nM of deoxy-nucleosides (dA, dT, dC, dG) for 24 h. Cells analyzed per condition > 12000. *P* value associated to two-sided *t*-test for the difference to the matched control. **c** The diagram of experimental settings for (**d, e**) is shown. **d** The induction of fork arrest by HU treatment in knockout MCF7 cells (CldU mean fork speed CAS = 0.29 kb/min, scored forks *N* = 582; *ATG5−*/*−* = 0.31 kb/min, *N* = 646; *ATG7−*/*−* = 0.18 kb/min, *N* = 703). *P* value associated to two-sided *t*-test with Welch’s correction. **e** Fork recovery after HU treatment in knockout MCF7 cells. CldU and HU were washed and cells were incubated for 20 min in the fresh medium containing IdU (IdU mean fork speed CAS = 0.48 kb/min, *N* = 582; *ATG5−*/*−* = 0.45 kb/min, *N* = 646; *ATG7−*/*−* = 0.26 kb/min, *N* = 703). *P* value associated to two-sided *t*-test with Welch’s correction. **f** The diagram of experimental settings for (**g**) is shown. **g** MCF7 CAS cells were pulse-labelled with CldU for 20 min, followed by a second pulse of IdU for 20 min. Before being pulse-labelled, cells were incubated with 100 nM of rapamycin for 6 h (Rapa), 2 nM of concanamycin A for 1.5 h (Conc. A) and 100 nM of dN for 1.5 h, where indicated. The length of CldU and IdU was measured and converted into fork speed in kb/min (results from two independent experiments; scored forks NT = 1405, average speed 1.28 kb/min; Rapa = 1209, 1.33 kb/min; Conc. A = 1274, 1.02 kb/min; NT + dN = 678, 1.21 kb/min; Rapa + dN = 546, 1.48 kb/min; Conc. A = 606, 1.33 kb/min). *P* value associated to two-sided *t*-test with Welch’s correction. **h** γH2AX mean nuclear intensity in T24 cells 72 h post transfection with siControl or siATG7 RNA. Cells analyzed per condition > 10000. *P* value associated to two-sided *t*-test for the difference to the matched control. **i** γH2AX mean nuclear intensity in T24 cells 72 h post transfection with siControl or siATG7 RNA. Cells analyzed per condition > 8000. *P* value associated to two-sided *t*-test for the difference to the matched control. **j** γH2AX mean nuclear intensity in PC-3 cells 72 h post transfection with siControl or siATG7 RNA. Cells analyzed per condition > 8000. *P* value associated to two-sided *t*-test for the difference to the matched control. **k** γH2AX mean nuclear intensity in PC-3 cells 72 h post transfection with siControl or siATG5 RNA. Cells analyzed per condition > 8000. *P* value associated to two-sided *t*-test for the difference to the matched control. **l** The level of the ATG7 protein was tested by immunoblotting in T24 cells 72 h after siRNA transfection. Actin was used as a loading control. **m** The level of the ATG5 protein was tested by immunoblotting in T24 cells 72 h after siRNA transfection. Actin was used as a loading control. **n** The level of the ATG7 protein was tested by immunoblotting in PC-3 cells 72 h after siRNA transfection. Actin was used as a loading control. **o** The level of the ATG5 protein was tested by immunoblotting in PC-3 cells 72 h after siRNA transfection. Actin was used as a loading control

Autophagy-deficient cells retained a high level of γH2AX and were more sensitive to HU compared with autophagy-proficient cells (Fig. 8a). If autophagy-deficient cells were more sensitive to nucleotide depletion, some of the phenotypes associated with RS could be alleviated by supplying cells with exogenous deoxy-nucleosides. Indeed, the level of γH2AX was reduced in autophagy-deficient cells after deoxy-nucleoside supplementation (Fig. 8b). Our results suggested that to some extent autophagy is necessary for optimal recovery from RS. Next, we tested specifically how DNA replication forks in autophagy-deficient cells respond to nucleotide depletion. Autophagy-deficient cells were incubated for 0.5 h with 2 mM of HU and for the last 20 min cells were pulse labelled with CldU. The distance travelled by CldU-labelled forks indicates the extent of fork progression vs arrest/delay. After CldU labelling, cells were washed and the fresh medium containing IdU was added for further 20 min. IdU-labelled forks indicate fork recovery after HU treatment (Fig. 8c). In the absence of *ATG5*, fork recovery was slightly impaired, while cells without *ATG7* showed more sensitivity to fork arrest and impaired recovery (Figs. 8d, e and S8A, B). To confirm that autophagy is necessary to sustain normal DNA synthesis, we have either induced autophagy with rapamycin or inhibited autophagy with concanamycin A and performed the DNA fibre assay (Fig. 8f). The induction of autophagy increased slightly fork speed without affecting fork symmetry (non-treated NT, fork seed 1.28 kb/min vs rapamycin-treated, fork speed 1.33 kb/min). In contrast, the inhibition of autophagy decreased significantly the speed of fork progression (concanamycin-treated, fork speed 1.0 kb/min) without affecting fork symmetry (Figs. 8g and S8C). Importantly, nucleoside supplementation alleviated the effect of concanamycin A (concanamycin + dN, fork speed 1.33 kb/min). Together, our results show that autophagy is required to maintain normal DNA synthesis and is important for recovery from RS.

Finally, relevant to our initial observation that autophagy is elevated in urinary bladder and prostate cancer samples, we depleted either *ATG5* or *ATG7* in a human bladder carcinoma cell line T24 and in a human prostate cancer cell line PC-3. In both cases, and analogous to the MCF7 and HeLa cells, the experimental reduction of these autophagy proteins resulted in the accumulation of RS markers, such as γH2AX and in less extend 53BP1 (Figs. 8h–o and S8D–G).

## Discussion

From a broader, conceptual perspective, our results can shed more light on the evolving topic of autophagy and cancer in two related, complementary ways. First, more generally, our data provide insights into the ongoing lively debate about the roles of autophagy during tumorigenesis, thereby complementing the reports on potential context-dependent involvement of autophagy as a tumour-suppressive vs tumour-promoting mechanism [14, 43, 44]. Second, at both conceptual and more mechanistic levels, we elucidate the relationship of autophagy with RS, both drug- and oncogene-induced, the latter emerging as a hallmark of cancer [45] that evokes the DDR checkpoint-mediated biological barrier against activated oncogenes and tumour progression [17, 18, 20].

With regard to the first issue, our present data from combined analyses of both human clinical material and cellular models with inducible oncogene expression, implicate autophagy in promotion, rather than suppression, of cancer. Our immunohistochemical analysis of autophagy markers on tissue specimens from different stages of human urinary bladder and prostate cancer progression showed features of activated autophagy in both early (Ta/T1-stage bladder and PIN lesions in prostate) and advanced lesions (invasive tumours), without any apparent decrease at advanced stages of disease progression. Overall, while the early appearance of autophagy markers is shared with DDR checkpoint activation [17, 18] and could possibly reflect activation of a tumour-suppressor barrier, the lack of autophagy mitigation in advanced lesions argues in favour of autophagy’s tumour-supporting role. In contrast to autophagy, markers of the DNA damage checkpoints, an established tumour-suppressor mechanism that also becomes activated in early human lesions, including the Ta urinary bladder stage, do become attenuated or lost in some advanced lesions [17]. This DDR checkpoint decline in subsets of advanced cancers reflects events, such as selection for loss-of-function mutations in the *ATM-Chk2* signalling module that activates p53, or selection for mutations in the *p53* tumour suppressor itself [17]. Our data also challenge the belief that autophagy might be activated exclusively to promote cellular senescence [46], a known tumour-suppressive mechanism, as we observed increased autophagy flux also in models, in which senescence is not induced by oncogenic activation (Fig. S3).

Overall to the first issue, while our results do not allow us to exclude the possibility that autophagy might somehow contribute to tumour suppression, we interpret our findings as largely consistent with the notion that autophagy supports tumour progression. The latter conclusion is also in concert with studies on murine cancer models [47] and it is further evident from our results on the relationship between autophagy and RS, as discussed below.

The second, arguably more innovative topic addressed in our present study is the mutual functional interplay between autophagy and RS. Whereas several studies have provided some insights into functional links between autophagy and DNA damage signalling and repair [43, 48–50], the interplay of autophagy with RS has so far remained understudied, despite this issue being intimately linked with cancer development and responses to chemotherapy. One important finding closely related to this topic, reported here, is the reproducible order of events in human cells, namely that in response to both oncogene- and drug-induced RS, the cellular DDR signalling machinery is activated earlier, followed by autophagy activation at a later stage. In our experiments, the lag period that separates DDR activation from the onset of autophagy was relatively short, ranging from several hours to maximally a few days. This may also explain why both DDR and autophagy activation appear to largely coincide in the early clinical lesions that we examined by immunohistochemistry, as these tissues, despite being collected from radio/chemotherapy-naive patients, were obviously obtained weeks, months or even years after the initial oncogenic event in vivo. Notably, our functional cell culture data are based on parallel analyses of several human cellular models and experiments with three different oncogenes commonly implicated in both pathogenesis and triggering of RS in a range of human cancers: H-Ras, c-Myc and cyclin E [45, 51–53]. While the precise kinetics of autophagy induction was, to some extent, oncogene-dependent, the overall pattern of DDR signalling preceding an autophagy flux increase was shared by all models used. We also employed RS-inducing drugs that are clinically used in cancer therapy, including HU and CPT, to further corroborate and extend the identified order of events ensuing the RS-inducing insult(s). The application of drugs further complements our data obtained with regulatable oncogenes in that the insult can be more precisely timed by adding the drug and removing it from the cell culture medium at desired time points. Indeed, combining an optimized high-content microscopy strategy to relate at the single-cell level the extent of DDR activity with autophagy activity during the cell cycle, we observed that upon short-term 30-min pulses of drug exposure of non-synchronized, exponentially growing human cell populations, only the subfraction of cells traversing through S phase and, hence, sensitive to the treatment during the short exposure period showed activated DDR signalling and the ensuing increase in autophagy flux. Furthermore, cells with the highest level of γH2AX after short-term RS-inducing treatments were the ones to feature the high amount of LC3B puncta in G2 phase, following drug-exposed S phase. On the other hand, we show that prolonged treatments with RS-inducing drugs lead to increased autophagy observed also in cells in G1 and G2 phases, with only little or no correlation between levels of DDR markers and autophagy flux. Such lack of correlation likely reflects the fact that when cells are exposed to genotoxic drugs for prolonged times, multiple cellular processes are impacted and diverse responses activated, making it difficult to establish any cause-consequence relationships. Collectively, the short-term drug exposure approach enabled us to exclude the possibility that autophagy is activated as a side effect by other cellular pathways than the immediate response to RS-triggered DDR.

Through a wide spectrum of functional analyses, including examination of replication fork speed and symmetry by DNA fibre assays as established “biomarkers” of the severity of RS, we addressed the impact on DNA synthesis and recovery from RS in human cell models that were genetically deprived of autophagy function through knockout of *ATG5* and *ATG7* genes, respectively. Our main results from these experiments lead to the following key conclusions, focusing on the relationship of autophagy with RS: (i) autophagy gene knockout triggers hallmarks of spontaneous RS and causes endogenous DNA damage; (ii) proficient autophagy is required for timely and efficient recovery of cells from drug-induced RS. Overall, our results indicate that autophagy plays a positive, cell-fitness-supporting role in preventing and/or coping with RS and that responses of autophagy-deficient human cells to mechanistically distinct types of RS are context dependent.

Furthermore, given the functional involvement of autophagy in cell metabolism, we also examined the impact of *ATG5/7* gene knockouts on key parameters of mitochondrial function and energy metabolism. Contrary to what has been observed in murine models [54], we show that autophagy loss did not result in altered levels of ROS or accumulation of defective mitochondria. Instead, we observed increased mitochondrial mass and, in some models, enhanced oxidative respiration and glycolysis, suggesting that cells might compensate autophagy loss by upregulating their energy metabolism. Indeed, plasticity of metabolic pathways may contribute to the emerging notion that manipulation of autophagy in clinical settings might not lead to desirable improvements in survival of cancer patients. Consistently, clinical trials using chloroquine or its derivatives have shown limited success [55, 56] and the cytotoxic effects of these drugs seem to be unrelated to autophagy inhibition [57, 58]. Another aspect of metabolism relevant to our present study is highlighted by aberrantly reduced speed of replication fork progression in human autophagy-deficient cells under both normal growth conditions and during recovery from HU treatment that depletes cellular nucleotides. These data suggested that autophagy might promote genomic stability and counteract RS, at least in part, by stabilizing nucleotide pools. This notion is also supported by our results that incubation of autophagy-deficient cell lines with 100 nM of exogenously supplied deoxy-nucleosides was sufficient to mitigate the extent of RS and rescue the phenotype of aberrantly enhanced, RS-associated γH2AX marker of DNA damage. Furthermore, autophagy inhibition decreased the speed of replication fork elongation, a phenomenon that was reversed by the addition of exogenous deoxy-nucleosides. Autophagy-regulatory mTOR signalling impacts nucleotide metabolism [59, 60] and autophagy may increase nucleotide levels through degradation of mRNA and ribosomes under certain stress conditions, thereby counteracting fluctuations in dNTP levels [54, 60]. Moreover, in yeast models, alkylation-induced DNA damage-induced autophagy specifically targets degradation of the RRM subunit RNR1, a process that favours the formation of the more efficient RNR1–RNR3 complex, rather than RNR1–RNR1, thus promptly promoting nucleotide pools under DNA damage conditions [61]. While consistent with our current results on RS and DNA damage, it remains to be seen whether an analogous mechanism operates also in mammalian cells.

Under normal conditions, the basal level of autophagy is needed to remove mis-folded proteins and damaged organelles to maintain cellular homoeostasis. Following starvation, autophagy is induced to recycle cellular components and supply building blocks for metabolic pathways [62]. Indeed, autophagy-deficient cells accumulate DNA damage, presumably by excessive degradation of checkpoint proteins [9] or perhaps by deregulated dNTP metabolism [63]. From the disease relevance point of view, our present results support the view that autophagy can support tumour progression, at least in part by responding to oncogene- or genotoxic therapy-induced RS that stimulates autophagy flux. In turn, enhanced autophagy helps to maintain fitness of the cancer cells, to better survive and cope with such stressful conditions. Our data also indicate that autophagy promotes optimal survival by stabilizing dNTP pools and sustaining DNA synthesis, and that the observed phenotypes are, to some extent, cell context-dependent. For example, the deoxy-nucleotide pools could differ among different cell types. For example, unlike in MCF7, the absence of autophagy did not affect replication forks considerably in the *ATG5/7-deficient* papilloma-virus-transformed HeLa cells. The latter aspect can also have therapeutic implications, since different types or subsets of tumours, or areas within a given tumour, that experience different degrees of RS, hypoxia, DNA damage, metabolic or proteotoxic stress, might consequently differ in their sensitivity or resistance to autophagy inhibition [64, 65]. Our results furthermore show that autophagy is needed for human cells to recover from RS, presumably by providing the cell with necessary amounts of metabolites required for repair and DNA synthesis. While this is a desirable effect in normal cells after cancer therapy, it is undesirable as a mechanism that can fuel tumorigenesis and resistance to standard-of-care genotoxic/RS-inducing treatments commonly used in oncology. Given that RS emerges as a hallmark feature shared by most, if not all, types of human malignancies [17, 20, 52], we would like to highlight the need to better understand the basic mechanisms of autophagy regulation and impact on normal vs transformed cells, to identify potential cancer cell vulnerabilities exploitable in treatment of cancer and possibly other chronic, aging-associated pathologies.

## Materials and methods

### Cell culture

Human BJ, U2-OS, SAOS, T24, PC-3 and HEK293 cell lines were purchased from ATCC. The U2-OS Cyclin E stable cell line overexpressing cyclin E under the control of a tetracycline response element was generated previously in our laboratory [18]. The U2-OS MycER stable cell line overexpressing the fusion protein MycER under a constitutive promoter was kindly provided by Prof Martin Eilers (University of Würzburg). The MCF7 and HeLa cell lines with knockout of *ATG5* and *ATG7* genes were generated by a standard lentiviral transduction procedure using plasmids kindly provided by Prof Kevin Ryan [66]. BJ, U2-OS and MCF7 cells with doxycycline (Dox) inducible expression of H-RasV12 (Lenti-X™ Tet-On Advanced Inducible Expression System, Clontech) were generated as described before [21].

U2-OS, SAOS, BJ, HEK293, T24, PC-3 and HeLa cell lines were maintained in DMEM (Gibco) supplemented with 10% FBS (Gibco) and penicillin/streptomycin (Gibco). The MCF7 cell line was grown in RPMI 1640 (Gibco) supplemented with 6% FBS and penicillin/streptomycin. U2-OS Cyclin E, U2-OS MycER, U2-OS H-RasV12, MCF7 H-RasV12 and BJ H-RasV12 were grown in DMEM without phenol red (DMEM/F-12, Gibco) supplemented with 10% FBS and penicillin/streptomycin. The medium for U2-OS Cyclin E cells was supplemented with 2 μg/ml of tetracycline. Cyclin E overexpression was induced by growing cells in the tetracycline-free medium. MycER translocation to the nucleus was induced with 100 nM of 4-hydroxytamoxifen. RAS overexpression was induced with 2 μg/ml of Dox.

### Chemicals

Cells were treated with different concentrations of the following drugs: aphidicolin (A0781, Sigma-Aldrich), camptothecin (CPT; C9911, Sigma-Aldrich), cisplatin (146262, Hospira), concanamycin A (27689, Sigma-Aldrich), deoxy-nucleosides (dA D8668, dG 854999, dT T1895, dC D3897, Sigma-Aldrich), hydroxyurea (HU; H-8627, Sigma-Aldrich), rapamycin (R0395, Sigma-Aldrich).

### Immunoblotting

Cells were washed twice with cold PBS and lysed in the 2x LSB buffer (4% SDS, 20% glycerol, 120 mM Tris-HCl pH 6.8). Cell extracts were separated by SDS-PAGE and transferred to the nitrocellulose or PVDF membranes (GE Healthcare). The membranes were blocked in 5% dry milk in 0.1% Tween-20 in PBS and probed with primary antibodies. After incubation with HRP-conjugated secondary antibodies (Vector Laboratories and Santa Cruz Biotechnology), proteins were visualized using ECL detection reagents (GE Healthcare). The primary antibodies used: phospho-ATM (S1981) (1:500, ab81292, Abcam), phospho-Chk2 (Thr68) (1:200, 2661, Cell Signalling), phospho-ATR (Ser428) (1:200, 2853, Cell Signalling), phospho-Chk1 (Ser345) (1:300, 2348, Cell Signalling), phospho-RPA32 (S33) (1:1000, A300-246A, Bethyl), gamma H2A.X (phospho-S140) (1:2000, ab22551, Abcam), H2AX (1:5000, NB100-638, Novus Biologicals), phospho-Rb (Ser807/811) (1:1000, 9308, Cell Signalling), p53 (DO-1) (1:1000, sc-126, Santa Cruz), p21 (H-164) (1:400, sc-756, Santa Cruz), phospho-p70 S6 Kinase (Thr389) (1:1000, 9206, Cell Signalling), p62 (1:1000, GP62-C, PROGEN Biotechnik), LC3B (D11) XP™ (1:1000, 3868, Cell Signalling), β-actin (1:10.000, A1978, Sigma-Aldrich), ATG5 (C-terminal) (1:700, A0731, Sigma-Aldrich), ATG7 (D12B11) (1:1000, 8558S, Cell Signalling).

### Immunofluorescence

Cells were grown on glass coverslips, washed with PBS and fixed with 4% formaldehyde for 10 min. After washing with PBS, cells were incubated with methanol at −20 °C for 10 min. Cells were then washed twice with PBS and incubated with primary antibodies for 1 h at RT. Following the washing step, coverslips were incubated with anti-rabbit or anti-mouse AlexaFluor-488 or -568 secondary antibodies (Invitrogen) with DAPI (Invitrogen) for 1 h at RT, washed again with PBS and mounted using Prolong Gold Antifade (Thermo Fisher Scientific). The primary antibodies used: LC3B (D11) XP™ (1:200, 3868, Cell Signalling), gamma H2A.X (phospho-S140) (1:1000, ab22551, Abcam), 53BP1 (H300) (1:500, sc-22760, Santa Cruz), TOMM20 (1:1000, ab56783, Abcam).

### EdU detection

Cells were incubated with 10 μM of EdU for 30 min prior to fixation. EdU staining was performed before incubation with primary antibodies by following manufacturer´s instruction for the Click-iT™ EdU Alexa Fluor™ 647 Imaging Kit (Thermo Fisher Scientific). Briefly, after permeabilization, coverslips were washed twice with 5% FBS in PBS and were incubated with the EdU staining solution for 30 min at RT (protected from the light). Afterwards, coverslips were washed once with 5% FBS in PBS and incubated with primary antibodies.

### High-content image acquisition

Quantitative image-based cytometry was performed as described previously [67]. Non-overlapping images were acquired in an unbiased and automated fashion with ScanR acquisition software and the Olympus ScanR microscope. Acquisition time was adjusted for each channel to avoid image saturation and at least 147 images were acquired for each condition. Automated focus was performed using the DAPI channel. Automated image analysis was performed with ScanR image analysis software. ScanR analysis results were exported as .txt files. The .txt dataset was then loaded into R software for further analysis.

### DNA fibre assay

Cells were pulse-labelled with 25 μM of CldU (Sigma-Aldrich) for 20 min, followed by a gentle wash with fresh prewarmed medium and the second pulse of 250 μM of IdU (Sigma-Aldrich) for 20 min. Cells were harvested and DNA fibres prepared as described previously [36]. CldU was detected with a rat anti-BrdU (OBT0030, Serotec) and a DyLight 550 anti-rat (Thermo Fisher Scientific) antibodies. IdU was detected with a mouse anti-BrdU (347580, Becton Dickinson) and the AlexaFluor-488 anti-mouse antibodies. Images of well-spread DNA fibres were acquired using an LSM800 confocal microscope (Carl Zeiss), a 63 × /1.4 oil immersion objective (Carl Zeiss) and LSM ZEN software. Analysis of double-labelled replication forks was performed manually using LSM ZEN software.

### Survival assay

Cells were treated with indicated drugs for different periods of time. The medium was then removed and cells were stained with Hoechst 33342 (1:2000, Invitrogen) and propidium iodide (1:1000, Invitrogen) in PBS for 15 min at RT. Each well was imaged with the aid of the Celigo Imaging Cytometer (Nexcelom Bioscience) following manufacturer’s instruction. To study the survival of cells after genotoxic stress, the number of dead cells (Hoechst and PI positive) in each well was subtracted from the total number of cells (Hoechst positive).

### ROS detection by flow cytometry

Cells were trypsinised, washed with PBS and resuspended in PBS (unstained), PBS with 5 µM of CellROX^TM^ Green (Thermo Fisher Scientific) (untreated) or PBS with 5 µM of CellROX^TM^ Green and 1 mM of H_2_O_2_ (Sigma-Aldrich) (H_2_O_2_). Cells were incubated for 45 min at 37 °C, loaded with 10 µg/ml of propidium iodide and analyzed immediately on FACSVerse (Becton Dickinson). Acquired data were analyzed using FlowJo software.

### ATP level analysis

ATP levels relative to parental cells were measured using the Luminescent ATP Detection Assay Kit (Abcam) following manufacturer’s instruction. Luminescence was measured with a FLUOstar Omega microplate reader (BMG Labtech).

### Seahorse analysis

Cells were seeded in a Seahorse XF96 Cell Culture microplate (Agilent) and a regular 96-well plate. OCR and ECAR were acquired using the Seahorse XFe96 analyzer following manufacturer’s instruction for the Seahorse XF Cell Mito Stress Test Kit. Briefly, a sensor cartridge in Seahorse XF Calibrant was hydrated overnight at 37 °C in a non-CO_2_ incubator and loaded with oligomycin (Port A), FCCP (Port B) and rotenone/antimycin-A (Port C). The medium in the Agilent Seahorse XF96 Cell Culture microplate was replaced with the Seahorse XF Base Medium supplemented with 10 mM of pyruvate, 2 mM of glutamine and 10 mM of glucose. The plate was then incubated for 1 h at 37 °C in the non-CO_2_ incubator. Calibration of the cartridge was then performed (15–30 min) and the calibration plate was replaced with the cell culture microplate before running the experiment. OCR and ECAR data were normalized for cell numbers by using the Celigo Imaging Cytometer as described above.

### Tumour tissue microarrays and patient information

We used two tissue microarrays to immunohistochemically analyze human bladder tumours collected at the Aarhus University Hospital (see [68] for additional patient information), one with 289 primary Ta/T1 early, non-invasive lesions from patients surgically treated by transurethral resection of the bladder (performed between 1979 and 2007), the other cohort of 425 specimens from patients with invasive tumours (stages T2–T4) operated by radical cystectomy (performed between 1992 and 2008). None of the patients included in the Ta/T1 cohort was treated by intravesical chemotherapy and the patients were regularly followed by control cystoscopies. For the patients whose tumours were included in the T2–T4 stage cohort, chemotherapy was administered only at the time of recurrence, not before the cystectomy. The tumours were classified and staged following the guidelines for grading from the World Health Organisation 2004 classification [69].

For the prostate cancer cohort, the tissue specimens were obtained from 35 patients (age range 51–81 years) operated by prostatectomies performed at the Palacky University Hospital in Olomouc, Czech Republic, between 2010 and 2011, and processed at the Pathology Department by standard formalin fixation to prepare paraffin blocks. Among this cohort, serum PSA levels were between 4 and 10 ng/ml for most (*n* = 23), while below 4 ng/ml in 4, and above 10 ng/ml in 8 patients, respectively. Gleason scores were 7 in 21 patients, below 7 in 8, and above 7 in 6 patients. The histopathological staging was performed by two experienced oncopathologists, and found to be of stage pT2a-c in 29 patients, pT3a-b in 5, and pT4 in 1 patient, respectively. From each of the 35 patients, areas of normal tissue (far from tumour), PIN and invasive PCa were identified. Informed consent was obtained from all patients in both research centres, and the studies were approved by the relevant ethical committees in Denmark and the Czech Republic, respectively.

### Immunohistochemistry

To detect the autophagy marker proteins and their patterns in human urinary bladder and prostate tissue and tumour specimens, we employed our well-established sensitive immunohistochemical staining protocol [17]. Formalin fixed, paraffin-embedded tissues were used from well-characterized tissue arrays, composed of human urinary bladder tissues and tumours of diverse grades (normal epithelium, early Ta–T1 stage lesions and advanced T2–T4 lesions) and the prostatic tissues and lesions (see the section above for tumour tissue microarrays, in which 195 and 308 of the Ta–T1 and T2–T4 stages for the bladder cancer array, respectively, along with all 35 prostate cancer standard paraffin tissue blocks were found to contain sufficiently representative tissue areas to be included in our present analysis). Standard deparaffinization of the archival formalin fixed, paraffin-embedded tissue sections was followed by antigen unmasking in the citrate buffer (pH 6, 15 min microwave exposure). After overnight incubation with primary antibodies, samples were processed for the indirect streptavidin–biotin–peroxidase method using the Vectastain Elite kit (Vector Laboratories) and nickel–sulfate-based chromogen enhancement detection as previously described, without nuclear counterstaining [17]. The primary antibodies used: rabbit polyclonal antibodies against LC3B (1:20000, NB100-2220, Novus Biologicals), p62 (1:10000, ab101266, Abcam) and LAMP-1 (1:5000, ab24170, Abcam). For negative controls, sections were incubated with non-immune rabbit serum. For positive controls, an antibody against human phospho-histone H2A.X (Ser 139) was used. Results were evaluated by a senior oncopathologist and data expressed in scoring categories based on the percentage of positive tumour cells expressing the respective protein. The degree of positivity for any of the autophagy proteins was scored in one of the four categories: A (0–5%), B (6–25%), C (26–75%) and D (76–100%) positive epithelial/carcinoma cells, respectively. According to the established procedures, the LC3B and p62 autophagy markers were scored for dot-like positivity in the cytoplasm and LAMP-1 for granular cytoplasmic positivity [26, 70]. While p62 staining was also separately scored for nuclear positivity [47, 71, 72], in our present study we focused on the “canonical” cytoplasmic staining.

## Supplementary information

Supplemental Figure S1

Supplemental Figure S2

Supplemental Figure S3

Supplemental Figure S4

Supplemental Figure S5

Supplemental Figure S6

Supplemental Figure S7

Supplemental Figure S8

supplementary figure legends
