# Supplementary figures and images for: Autophagy role(s) in response to oncogenes and DNA replication stress
Source: Cell Death Differ. 2019 Aug 14;27(3):1134–53. doi: 10.1038/s41418-019-0403-9 (PMC7206042; doi:10.1038/s41418-019-0403-9)

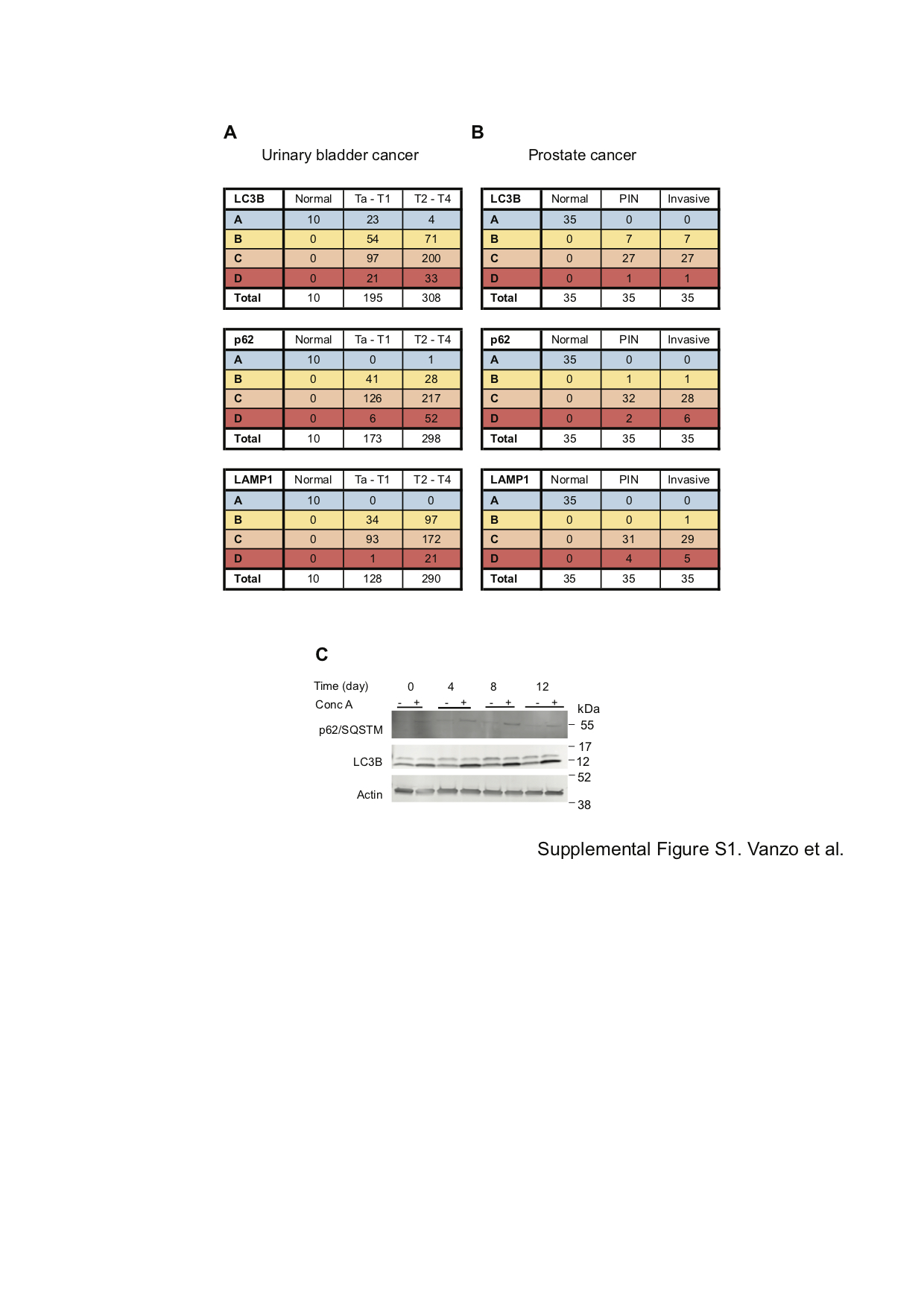

Supplement: Supplementary file 1 — Supplemental Figure S1 [file 41418_2019_403_MOESM1_ESM.jpg]

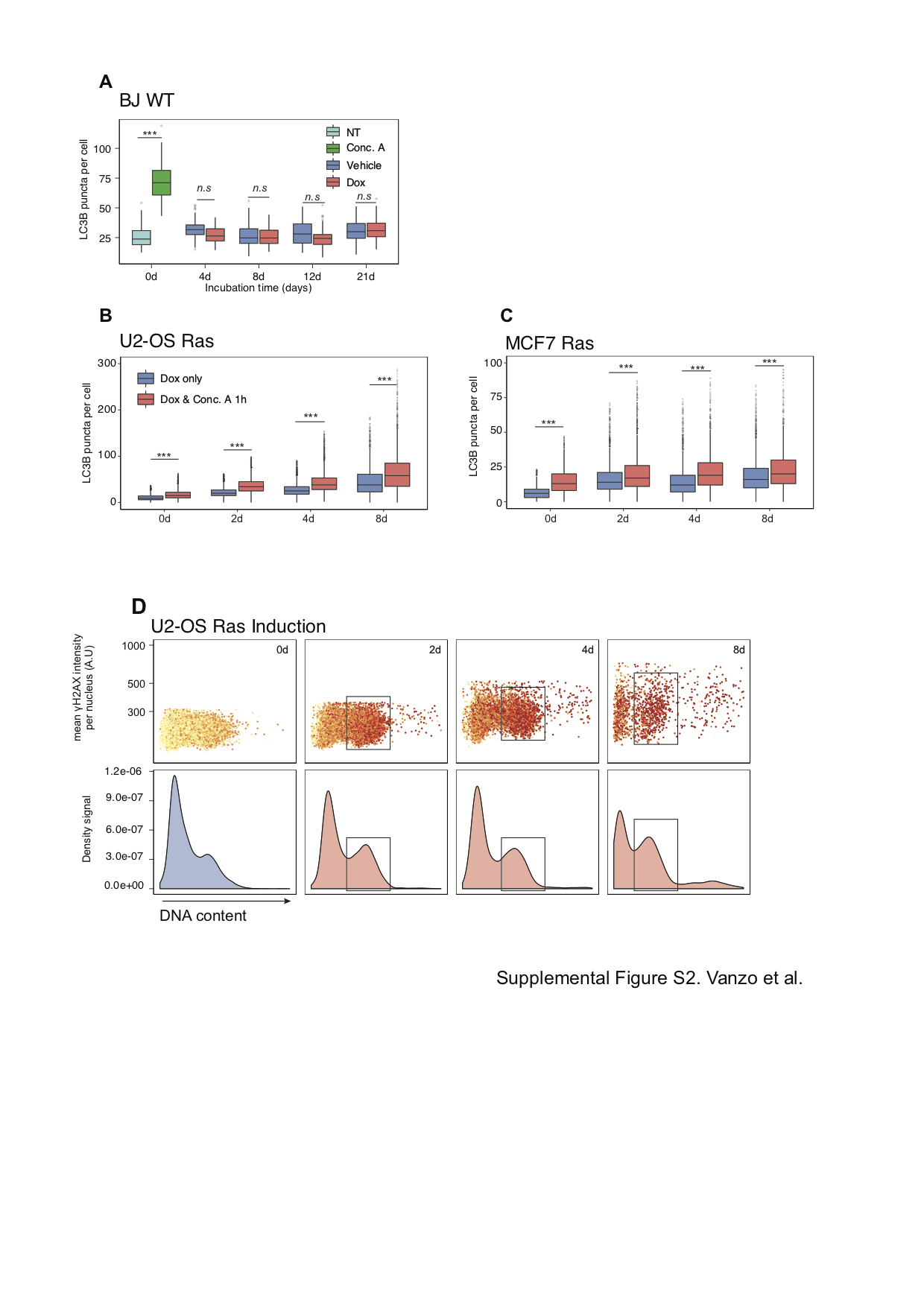

Supplement: Supplementary file 2 — Supplemental Figure S2 [file 41418_2019_403_MOESM2_ESM.jpg]

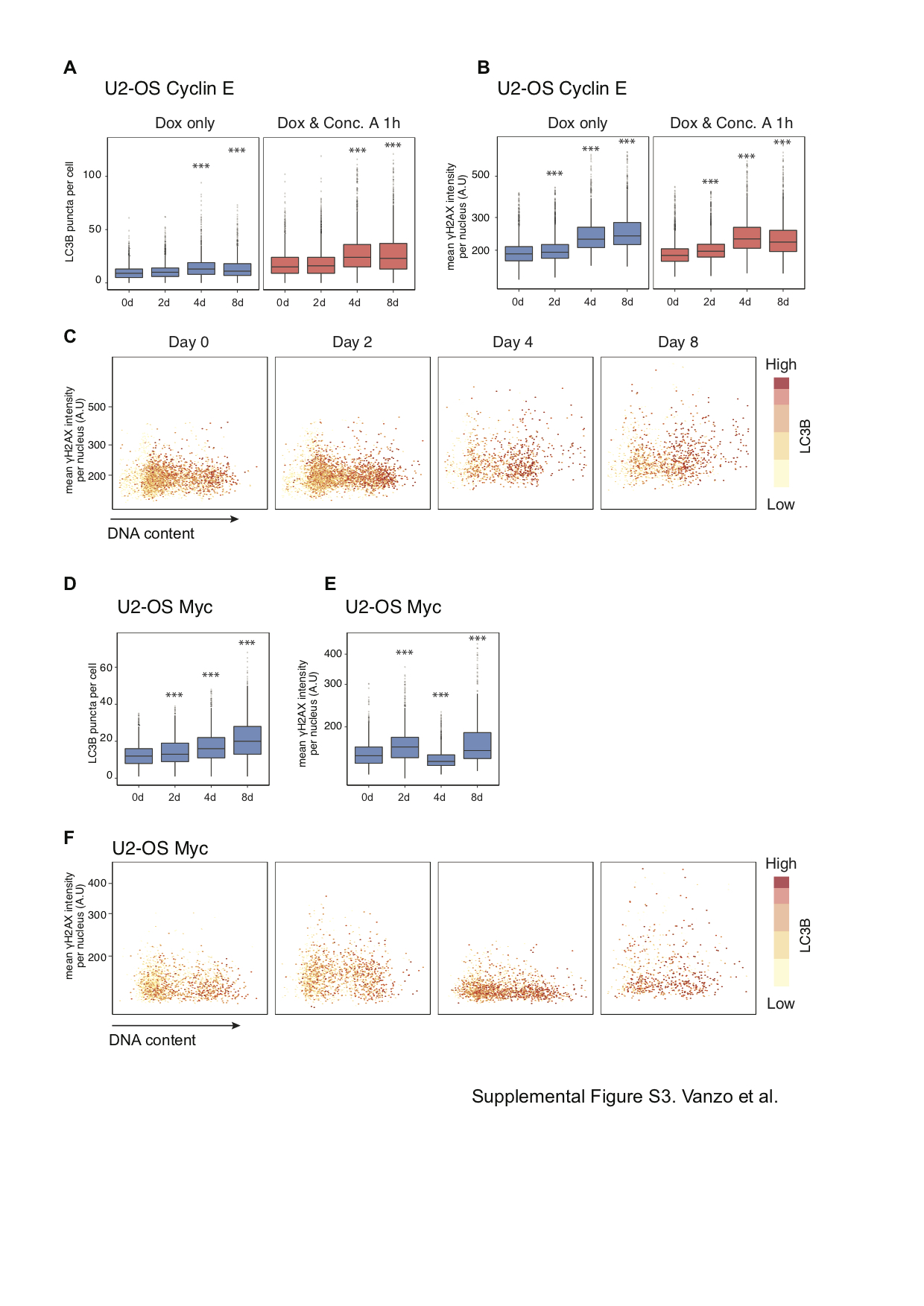

Supplement: Supplementary file 3 — Supplemental Figure S3 [file 41418_2019_403_MOESM3_ESM.jpg]

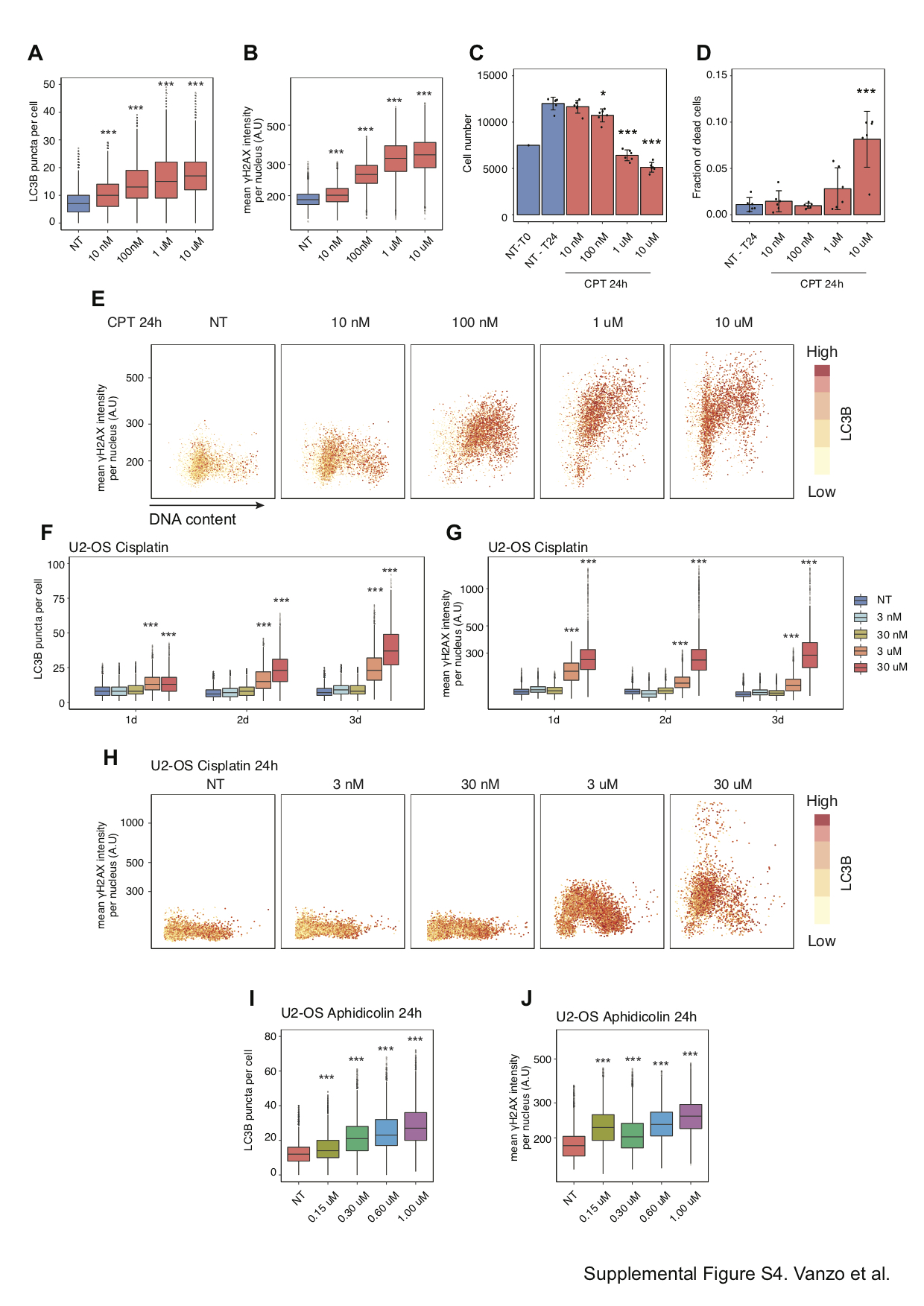

Supplement: Supplementary file 4 — Supplemental Figure S4 [file 41418_2019_403_MOESM4_ESM.jpg]

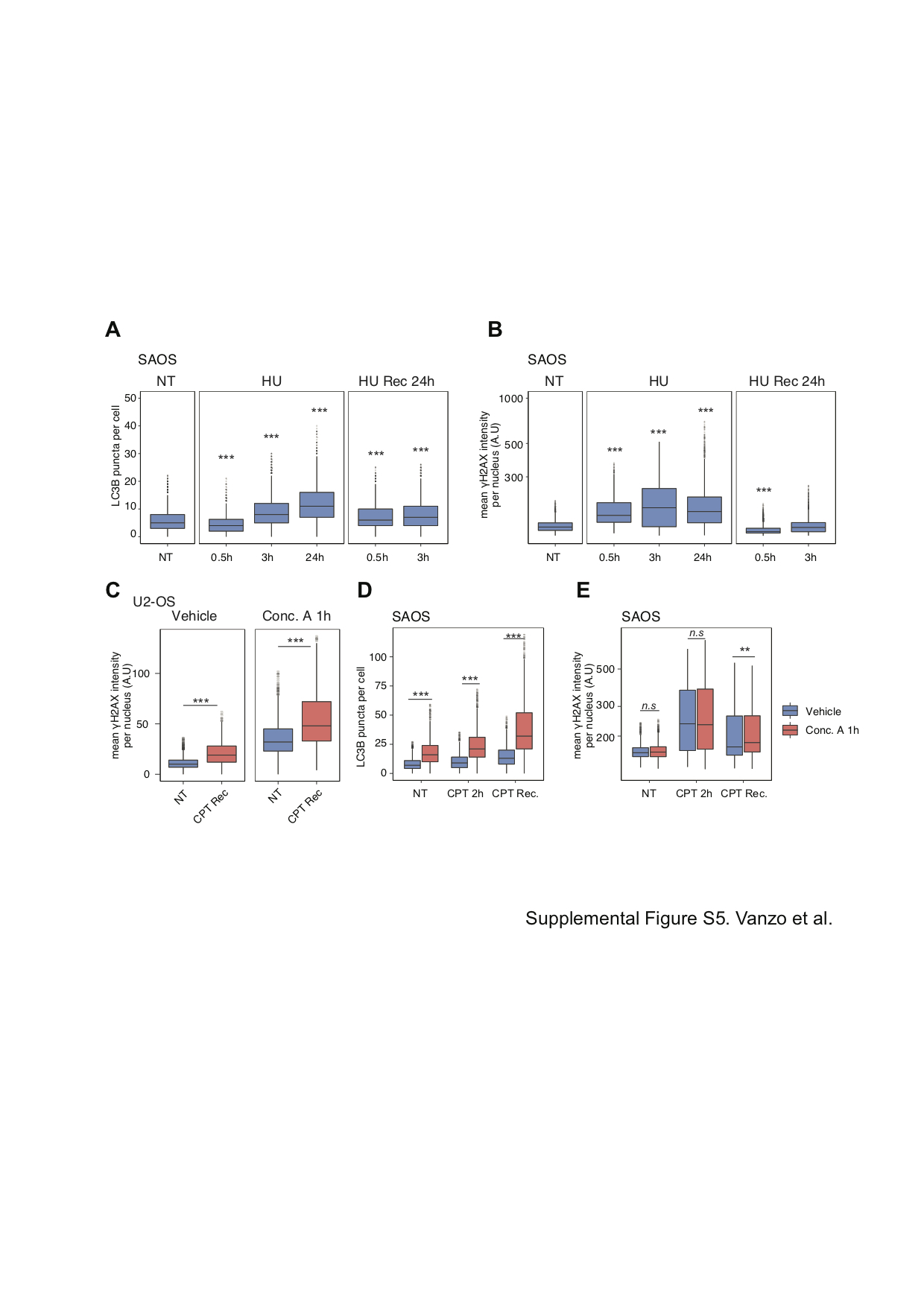

Supplement: Supplementary file 5 — Supplemental Figure S5 [file 41418_2019_403_MOESM5_ESM.jpg]

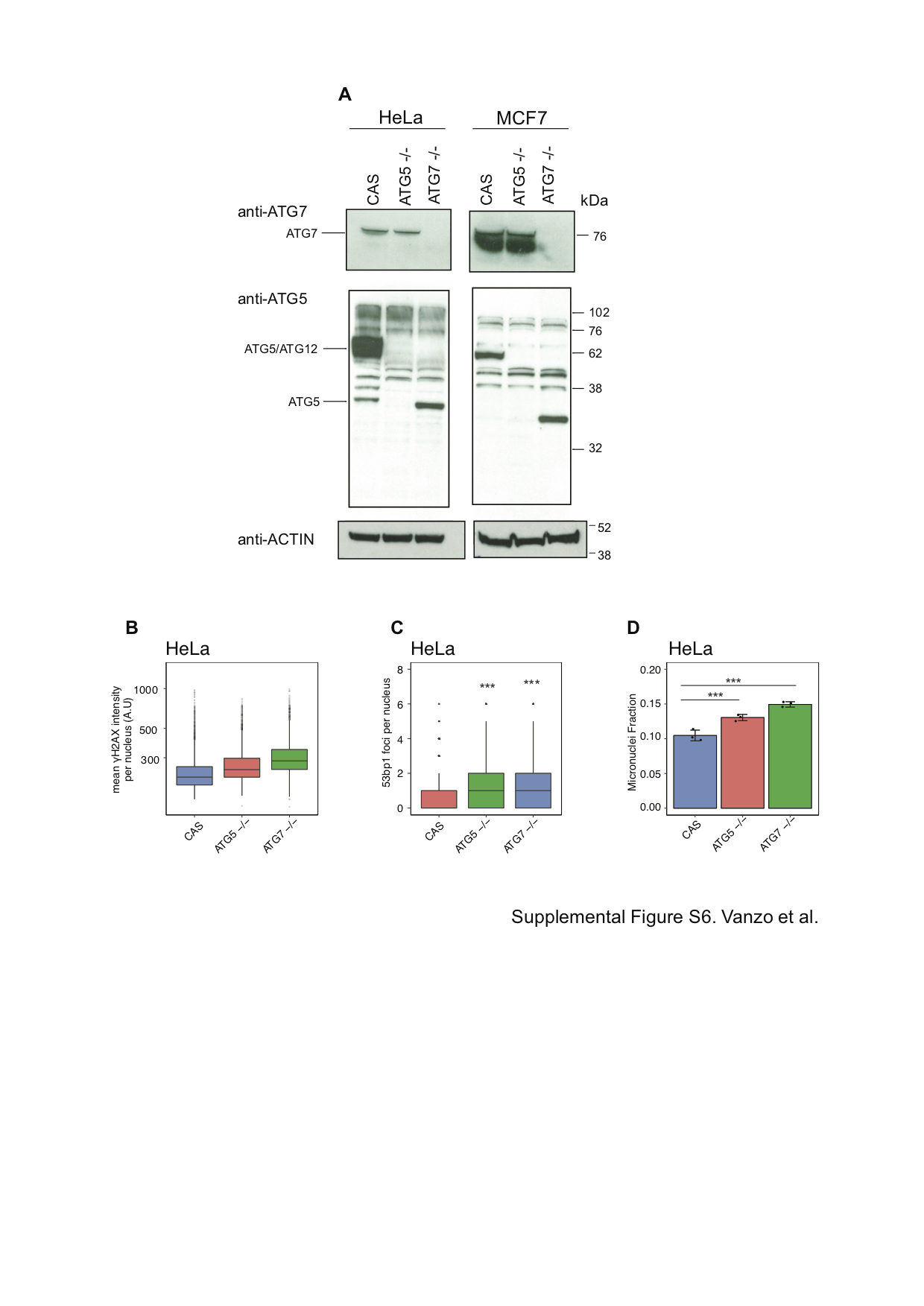

Supplement: Supplementary file 6 — Supplemental Figure S6 [file 41418_2019_403_MOESM6_ESM.jpg]

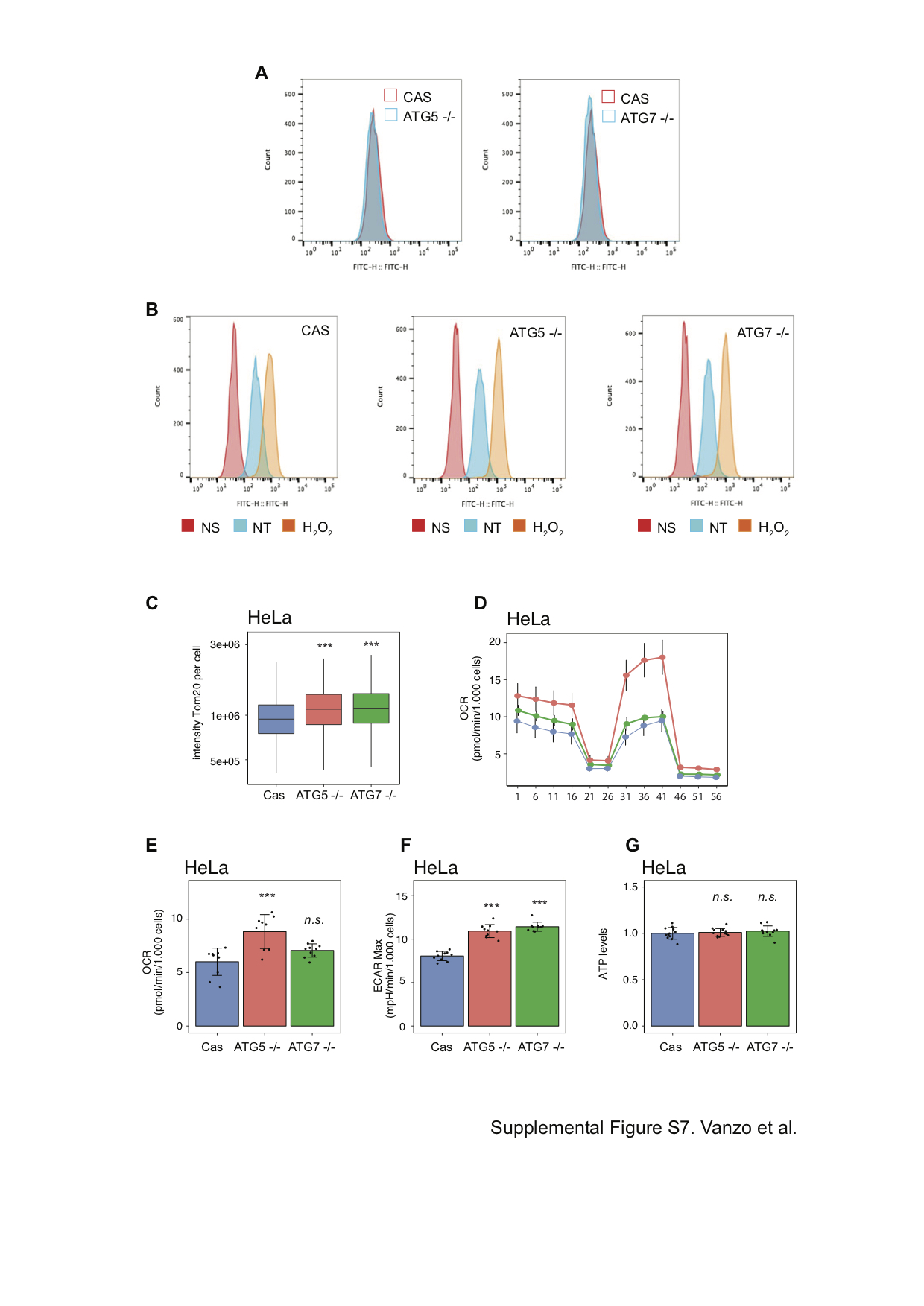

Supplement: Supplementary file 7 — Supplemental Figure S7 [file 41418_2019_403_MOESM7_ESM.jpg]

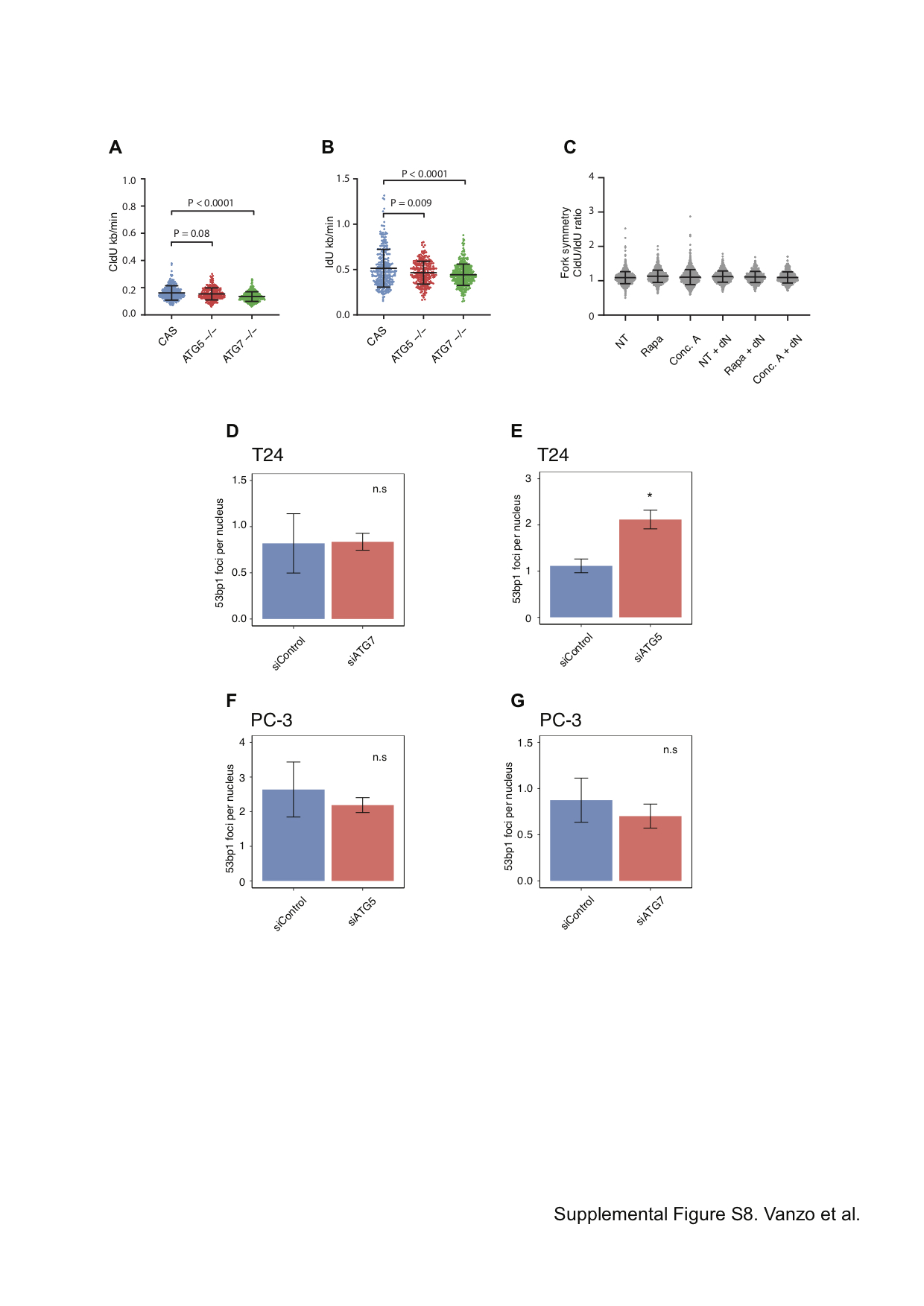

Supplement: Supplementary file 8 — Supplemental Figure S8 [file 41418_2019_403_MOESM8_ESM.jpg]
