# Supplementary material for: Autophagy role(s) in response to oncogenes and DNA replication stress
Source: Cell Death Differ. 2019 Aug 14;27(3):1134–53. doi: 10.1038/s41418-019-0403-9 (PMC7206042; doi:10.1038/s41418-019-0403-9)
Supplement: Supplementary file 9 — supplementary figure legends [file 41418_2019_403_MOESM9_ESM.docx]

**Supplemental Figure legends**

**Supplemental Figure S1.** **Autophagy is activated during tumorigenesis.** (**A**) The total number of samples analyzed for bladder cancer. (**B**) The total number of samples analyzed for prostate cancer. (**C**) BJ-Ras cells were treated with 2 μg/ml of Doxycycline (Dox) for the indicated time. Where indicated, cells were incubated with 2 nM of Concanamycin A for 1 h prior to lysis and analyzed by immunoblotting. Actin was used as a loading control.

**Supplemental Figure S2.** **H-RasV12 overexpression induces autophagy.** (**A**) Quantification of LC3B puncta per cell in WT BJ fibroblasts. Cells were incubated with DMSO (Vehicle) or 2 μg/ml of Doxycycline (Dox) for the indicated days. As a positive control of an increased amount of autophagosomes, BJ fibroblast were incubated for 1 h with 2 nM of Concanamycin A (Conc. A). Pictures analyzed per condition > 200, at least 20 cells per picture. *P* value associated to two-sided *t*-test for the difference to the non-treated control. (**B**) Quantification of LC3B puncta per cell in U2-OS cells incubated with 2 μg/ml of Doxycycline (Dox) for the indicated days. Where indicated, cells were incubated with 2 nM of Concanamycin A (Conc. A) for 1 h prior to fixation. Cells analyzed per condition > 1500. *P* value associated to two-sided *t*-test for the difference to the non-treated control. (**C**) Quantification of LC3B puncta per cell in MCF7 cells incubated with 2 μg/ml of Doxycycline (Dox) for the indicated days. Where indicated, cells were incubated with 2 nM of Concanamycin A (Conc. A) for 1 h prior to fixation. Cells analyzed per condition > 1500. *P* value associated to two-sided *t*-test for the difference to the non-treated control. (**D**) Upper row: A single-cell analysis (single points on the scattering plot) of γH2AX mean nuclear intensity (y-axis), LC3B puncta per cell (color code) and DNA content (x-axis) from Figure 2 F. Lower row: A density plot of DAPI total intensity (cell cycle profile) for the matched sample. Squares indicate late-S/G2 cell populations.

**Supplemental Figure S3.** **Cyclin E or c-MYC overexpression induces autophagy.** (**A**) Quantification of LC3B puncta per cell in U2-OS cells containing the Cyclin E expression vector. Cells at days 0-8 were maintained in the DMEM medium supplemented with 2 μg/ml of Doxycycline (Dox). Where indicated, cells were incubated with 2 nM of Concanamycin A (Conc. A) for 1 h prior to fixation. Cells analyzed per condition > 1000. *P* value associated to two-sided *t*-test for the difference to the non-treated control. (**B**) Quantification of γH2AX mean nuclear intensity per cell in U2-OS cells containing the Cyclin E expression vector, with experimental conditions as in (**A**). *P* value associated to two-sided *t*-test for the difference to the non-treated control. (**C**) A single-cell analysis of γH2AX mean nuclear intensity, LC3B puncta per cell and DNA content in U2-OS cells containing the Cyclin E expression vector, with experimental conditions as in (**A**). Cells analyzed per condition > 1000. (**D**) Quantification of LC3B puncta per cell in U2-OS cells containing the Myc-ER inducible expression vector. Cells were incubated with 100 nM of 4-hydroxytamoxifen for the indicated time. Cells analyzed per condition > 650. *P* value associated to two-sided *t*-test for the difference to the non-treated control. (**E**) Quantification of γH2AX mean nuclear intensity per cell in U2-OS cells containing the Myc-ER inducible expression vector, with experimental conditions as in (**D**). *P* value associated to two-sided *t*-test for the difference to the non-treated control. (**F**) A single-cell analysis of γH2AX mean nuclear intensity, LC3B puncta per cell and DNA content in U2-OS cells containing the Myc-ER inducible expression vector, with experimental conditions as in (**D**). Cells analyzed per condition > 1000.

**Supplemental Figure S4.** **Drugs that induce DNA replication stress trigger autophagy.** (**A**) Quantification of LC3B puncta per cell in U2-OS cells incubated with the indicated concentrations of Camptothecin (CPT) for 24 h. Cells analyzed per condition > 3500. *P* value associated to two-sided *t*-test for the difference to the non-treated control. (**B**) γH2AX mean nuclear intensity in cells treated as in (**A**). Cells analyzed per condition > 3500. *P* value associated to two-sided *t*-test for the difference to the non-treated control. (**C**) Quantification of U2-OS cells incubated for 24 h with the indicated concentrations of Camptothecin (CPT). Error bars indicate mean and SD for each independent biological replicate (N = 6). *P* value associated to two-sided *t*-test for the difference to the untreated control. (**D**) Quantification of the fraction of dead U2-OS cells from (**C**). Error bars indicate mean and SD for each independent biological replicate (N = 6). (**E**) A single-cell analysis of γH2AX mean nuclear intensity, LC3B puncta per cell and DNA content in U2-OS cells treated as in (**A**). (**F**) Quantification of LC3B puncta per cell in U2-OS cells incubated with the indicated concentrations of Cisplatin for 24 h. Cells analyzed per condition > 1500. *P* value associated to two-sided *t*-test for the difference to the non-treated control. (**G**) γH2AX mean nuclear intensity in cells treated as in (**F**). Cells analyzed per condition > 1500. *P* value associated to two-sided *t*-test for the difference to the non-treated control. (**H**) A single-cell analysis of γH2AX mean nuclear intensity, LC3B puncta per cell and DNA content in U2-OS cells treated as in (**F, G**). (**I**) Quantification of LC3B puncta per cell in U2-OS cells incubated with the indicated concentrations of Aphidicolin for 24 h. Cells analyzed per condition > 7000. *P* value associated to two-sided *t*-test for the difference to the non-treated control. (**J**) γH2AX mean nuclear intensity in cells treated as in (**I**). Cells analyzed per condition > 7000. *P* value associated to two-sided *t*-test for the difference to the non-treated control.

**Supplemental Figure S5.** **Induction of autophagy in p53-deficient SAOS cells.** (**A**) Quantification of LC3B puncta per cell in SAOS cells. Where indicated, cells were treated with 2 mM of Hydroxyurea (HU) for the indicated time, washed and left for 24 h to recover. Cells analyzed per condition > 4000. *P* value associated to two-sided *t*-test for the difference to the non-treated control. (**B**) γH2AX mean nuclear intensity in cells treated as in (**A**). *P* value associated to two-sided *t*-test for the difference to the non-treated control. (**C**) Quantification of LC3B puncta per cell in U2-OS cells. Where indicated, cells were treated with 1 μM of Camptothecin (CPT) for 2 h, washed and left for 24 h to recover (CPT Rec). Some cells were also incubated with 2 nM of Concanamycin A (Conc. A) for 1 h prior to fixation. Cells analyzed per condition > 1000. *P* value associated to two-sided *t*-test for the difference to the non-treated control. (**D**) Quantification of LC3B puncta per cell in SAOS cells. Where indicated, cells were treated with 1 μM of Camptothecin (CPT) for 2 h, washed and left for 24 h to recover (CPT Rec). Some cells were also incubated with 2 nM of Concanamycin A (Conc. A) for 1 h prior to fixation. Cells analyzed per condition > 1500. *P* value associated to two-sided *t*-test for the difference to the non-treated control. (**E**) γH2AX mean nuclear intensity in cells treated as in (**D**). *P* value associated to two-sided *t*-test for the difference to the non-treated control.

**Supplemental Figure S6. Autophagy-deficient HeLa cells accumulate replication stress.** (**A**) Total protein extracts from knockout MCF7 and HeLa cells were examined by immunoblotting for the levels of ATG5 and ATG7. Actin was used as a loading control. (**B**) γH2AX mean nuclear intensity per nucleus in knockout HeLa cells: (CAS) parental control, *ATG5*-/- and *ATG7*-/-. Cells analyzed per condition > 8000. (**C**) An average number of 53BP1 foci per nucleus in knockout HeLa cells: (CAS) parental control, *ATG5*-/- and *ATG7*. Cells analyzed per condition > 80000. *P* value associated to two-sided *t*-test for the difference to the matched control. (**D**) Micronuclei fraction in knockout HeLa cells: (CAS) parental control, *ATG5*-/- and *ATG7*-/- (N=3). *P* value associated to two-sided *t*-test for the difference to the matched control.

**Supplemental Figure S7. Knock-out of autophagy genes in HeLa cells has no detrimental effect on basal metabolism**. (**A**) The level of ROS was measured by flow cytometry in *ATG5-* and *ATG7*-knockout HeLa cells. (**B**) As a positive control of ROS production, cells were treated with 1 mM of H_2_O_2_ for 45 min (NS, non-stained; NT, non-treated; H_2_O_2_, H_2_O_2_-treated). (**C**) Tom20 mean intensity per cell in knockout HeLa cells: (CAS) parental control, *ATG5*-/- and *ATG7*-/-. Cells analyzed per condition > 9000. *P* value associated to two-sided *t*-test for the difference to the matched control. (**D**) Oxygen consumption rate (OCR) in *ATG5*- and *ATG7*-knockout HeLa cells. Cells were analyzed as in Figure 7 B. Samples analyzed per cell line N=8. (**E**) The mean of OCR values for knockout HeLa cells. Samples analyzed per cell line N=8. *P* value associated to two-sided *t*-test for the difference to the matched control. (**F**) The mean of ECAR values for knockout HeLa cells. OCR and ECAR values were corrected for non-mitochondrial respiration. Samples analyzed per cell type N=8. *P* value associated to two-sided *t*-test for the difference to the matched control. (**G**) The mean ATP level in knockout HeLa cells (N=12). *P* value associated to two-sided *t*-test for the difference to the matched control.

**Supplemental Figure S8. Autophagy is required for efficient recovery from RS in HeLa cells.** (**A**) The induction of fork arrest by HU treatment as in Figure 8C using knockout HeLa cells (CldU mean fork speed CAS=0.16 kb/min, scored forks N=294; *ATG5*-/-=0.15 kb/min, N=249; *ATG7*-/-=0.13 kb/min, N=378). *P* value associated to Kolmogorov-Smirnov test. (**B**) Fork recovery after HU treatment in knockout HeLa cells. CldU and HU were washed and cells were incubated for 20 min in the fresh medium containing IdU (IdU mean fork speed CAS=0.51 kb/min, N=294; *ATG5*-/-=0.46 kb/min, N=249; *ATG7*-/-=0.44 kb/min, N=378). *P* value associated to Kolmogorov-Smirnov test. (**C**) MCF7 CAS cells were pulse-labelled with CldU for 20 min, followed by a second pulse of IdU for 20 min. Before being pulse-labelled, cells were incubated with 100 nM of Rapamycin for 6h (Rapa), 2 nM of Concanamycin A for 1.5 h (Conc. A) and 100 nM of dN for 1.5 h, where indicated. The ratio between CldU/IdU was analyzed and plotted as relative frequencies. (**D**) 53BP1 foci per cell in T24 cells 72 h post-transfection with siControl or siATG5 RNA. Cells analyzed per condition > 10000. *P* value associated to two-sided *t*-test for the difference to the matched control. (**E**) 53BP1 foci per cell in T24 cells 72 h post-transfection with siControl or siATG7 RNA. Cells analyzed per condition > 8000. *P* value associated to two-sided *t*-test for the difference to the matched control. (**F**) 53BP1 foci per cell in PC-3 cells 72 h post-transfection with siControl or siATG5 RNA. Cells analyzed per condition > 8000. *P* value associated to two-sided *t*-test for the difference to the matched control. (**G**) 53BP1 foci per cell in PC-3 cells 72 h post-transfection with siControl or siATG7 RNA. Cells analyzed per condition > 8000. *P* value associated to two-sided *t*-test for the difference to the matched control.
